# RBMS3-induced circHECTD1 encoded a novel protein to suppress the vasculogenic mimicry formation in glioblastoma multiforme

**DOI:** 10.1038/s41419-023-06269-y

**Published:** 2023-11-15

**Authors:** Xuelei Ruan, Yunhui Liu, Ping Wang, Libo Liu, Teng Ma, Yixue Xue, Weiwei Dong, Yubo Zhao, Tiange E, Hongda Lin, Di Wang, Chunqing Yang, Jian Song, Jiate Liu, Meiqi Deng, Ping An, Yang Lin, Jin Yang, Zheng Cui, Yaming Cao, Xiaobai Liu

**Affiliations:** 1 Department of Neurobiology, School of Life Sciences, China Medical University, Shenyang, 110122 China; 2 Key Laboratory of Neuro-oncology in Liaoning Province, Shenyang, 110004 China; 3 Department of Neurosurgery, Shengjing Hospital of China Medical University, Shenyang, 110004 China; 4 Department of Immunology, College of Basic Medical Sciences, China Medical University, Shenyang, 110122 Liaoning China

**Keywords:** CNS cancer, Non-coding RNAs, Mechanisms of disease, Tumour-suppressor proteins

## Abstract

Glioblastoma multiforme (GBM) is a highly vascularized malignant cancer of the central nervous system, and the presence of vasculogenic mimicry (VM) severely limits the effectiveness of anti-vascular therapy. In this study, we identified downregulated circHECTD1, which acted as a key VM-suppressed factor in GBM. circHECTD1 elevation significantly inhibited cell proliferation, migration, invasion and tube-like structure formation in GBM. RIP assay was used to demonstrate that the flanking intron sequence of circHECTD1 can be specifically bound by RBMS3, thereby inducing circHECTD1 formation to regulate VM formation in GBM. circHECTD1 was confirmed to possess a strong protein-encoding capacity and the encoded functional peptide 463aa was identified by LC-MS/MS. Both circHECTD1 and 463aa significantly inhibited GBM VM formation in vivo and in vitro. Analysis of the 463aa protein sequence revealed that it contained a ubiquitination-related domain and promoted NR2F1 degradation by regulating the ubiquitination of the NR2F1 at K396. ChIP assay verified that NR2F1 could directly bind to the promoter region of MMP2, MMP9 and VE-cadherin, transcriptionally promoting the expression of VM-related proteins, which in turn enhanced VM formation in GBM. In summary, we clarified a novel pathway for RBMS3-induced circHECTD1 encoding functional peptide 463aa to mediate the ubiquitination of NR2F1, which inhibited VM formation in GBM. This study aimed to reveal new mechanisms of GBM progression in order to provide novel approaches and strategies for the anti-vascular therapy of GBM.

**Figure:**
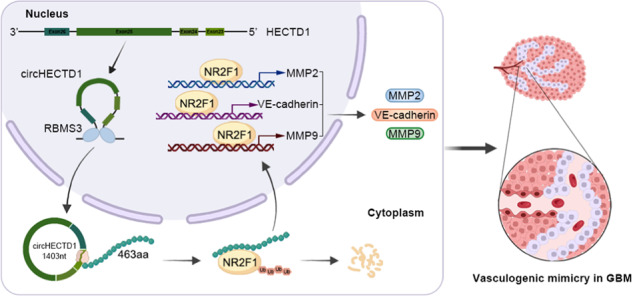
The schematic illustration showed the inhibitory effect of circHECTD1-463aa in the VM formation in GBM.

The schematic illustration showed the inhibitory effect of circHECTD1-463aa in the VM formation in GBM.

## Introduction

Extreme invasiveness and excessive vascularization are hallmarks of glioblastoma multiforme (GBM), which is the most aggressive primary tumor of the central nervous system (CNS) [1]. The traditional comprehensive treatment programs such as surgical resection combined with radiotherapy, chemotherapy, or anti-vascular therapy, have shown inefficiency and poor prognosis. The 5-year survival rate of GBM patients is only 6.8% [2, 3]. Studies have shown that abundant blood vessels and blood supply in GBM are essential factors for refractory and recurrent GBM [4]. On the one hand, the brain vascular endothelial cells can form new blood vessels by sprouting or other mechanisms [5]. On the other hand, GBM cells can create a lumen-like structure that is surrounded by periodic acid-Schiff (PAS)-positive staining basement membrane in endothelial cells independent manner to afford adequate blood furnish for GBM growth, termed as vasculogenic mimicry (VM) [6]. Emerging evidence suggests that VM plays a crucial regulatory role in the occurrence and development of GBM [7]. Therefore, an in-depth study of the mechanism of VM formation is a vital approach to find new targets for GBM anti-vascular treatment.

Circular RNAs (circRNAs) have been reported to play an important role in regulating VM formation in multiple tumors. Studies have shown that exosomal circRNA-100338 in hepatocellular carcinoma alters VM formation capacity by regulating VE-cadherin [8]. Targeting circDGKD intercepts VM formation in renal cell carcinoma induced by sunitinib treatment and improves survival in nude mice [9] However, little is known about the mechanisms by which circRNAs regulate VM formation in GBM. Circular RNAs are mostly produced via back-splicing of precursor mRNA (pre-mRNA) exons and are distinguished by their covalently closed loop structures without a 5′ cap or a 3′ Poly A tail [10, 11]. circRNAs are not easily degraded by exonucleases and exhibit tissue-specific or cell-type-specific patterns [12]. In GBM, dysregulated circRNAs reportedly participate in various stages of tumor progression. Previously, we jointly analyzed two GSE datasets containing the circRNAs expression profiles in GBM, and combined with qPCR assays to screen out circHECTD1 (hsa_circ_0002301), which is significantly downregulated in GBM.

In recent years, circRNAs, as non-coding RNAs, have stimulated the interest of researchers owing to the new peptide-coding functions [13]. Studies have shown that circ-FBXW7 encodes a 21 kDa functional peptide through its open reading frame (ORF), which shows an inhibitory effect on cell proliferation and malignant phenotype in GBM [14]. circPPP1R12A-73aa, a novel circPPP1R12A-encoded protein, induces tumor metastasis and pathogenesis in colon cancer [15]. Analysis of the circRNADb database reveals that circHECTD1 contains ORF and internal ribosome entry site (IRES) components, and thus may perform encoding functions. Therefore, our study intends to investigate whether circHECTD1, which is lowly expressed in GBM tissues, regulated VM formation in GBM through its potential coding function.

RNA binding motif single stranded interacting protein 3 (RBMS3), encodes an RNA-binding protein (RBP) with glycine-rich, and belongs to the c-Myc single-strand binding protein family [16]. RBMS3 participates in modulating several biological processes, consisting of cell apoptosis, gene transcription, cell cycle progression, etc. [17]. Moreover, RBMS3 plays an essential regulatory role as a tumor suppressor gene in a variety of tumors. For instance, RBMS3 is downregulated in gastric cancer, gallbladder cancer, and esophageal squamous cell carcinoma, and its low expression is positively associated with poor prognosis [18–20].

Nuclear receptor subfamily 2 group F member 1 (NR2F1), a nuclear receptor of the steroid/thyroid hormone receptor superfamily, acts as a primary transcriptional regulator of processes such as cortical regionalization, cell-specific specification, and maturation [21]. NR2F1 promotes the proliferative and migration capacity in breast cancer cells through the CXCL12/CXCR4 pathway and also enhances the ability of salivary gland adenoid cystic carcinoma cells to invade and metastasize [22, 23]. However, the mechanism by which NR2F1 regulates VM in GBM cells remains unexplored.

Cadherin 5 (CDH5), also known as VE-cadherin, is highly expressed in GBM and positively correlates with patients’ poor prognosis. Its high expression can induce VM channel formation in glioblastoma stem cells, and VE-cadherin is a recognized molecular marker of VM formation [24]. Matrix metallopeptidase 9 (MMP9) and matrix metallopeptidase 2 (MMP2) are the most frequent metalloproteases associated with tumor angiogenesis [25]. Studies have shown that MMP2 and MMP9 activation can increase the expression of laminin subunits in the cell matrix membrane, leading to the remodeling of tumors’ extracellular matrix, and thereby promoting VM formation [26]. MMP2 and MMP9 are also highly expressed in GBM, and the downregulation of MMP2 and MMP9 significantly inhibits VM in GBM cells [27].

In this study, endogenous expression of RBMS3, circHECTD1, and NR2F1 in GBM cells and tissues was first identified. The relationships among these factors were further investigated to clarify their effects on VM formation, including GBM cell proliferation, migration, invasion, and tube-forming capacity. The study aimed to provide a new mechanism for GBM progression as well as novel approaches and strategies for the anti-vascular therapy of GBM.

## Materials and methods

### Cell culture and human tissue samples

Details of the tissue samples and cells used in the study were described in Supplementary File 1.

### Quantitative Real-Time PCR

Trizol reagent (Invitrogen, USA) was applied to extract RNA from GBM cells with different treatments and adjusted to the same concentration (50 ng/μL). One-Step SYBR Prime Script RT-PCR Kit (RR064A, TakaraBio, Japan) was used to measure the relative expression levels of circHECTD1, NR2F1, MMP2, MMP9, VE-cadherin and HECTD1, with the help of Applied Biosystems 7500 Fast RT-PCR System. GAPDH and U6 were used as endogenous control. The fold change was calculated in accordance with the relative quantification (2^−ΔΔCt^) method. The amplification conditions were followed: 42 °C for 5 min and 95 °C for 10 s respectively for 1 reps, further 40 reps of 95 °C for 5 s and 60 °C for 34 s. The related primer sequence was listed in Supplemetary table 1.

### RNA fluorescence in situ hybridization (FISH) assay

SA-Cy3-labeled circHECTD1 probe was designed and synthesized (GenePharma, China). The probe signals were determined by the FISH Kit with the SA-Biotin system (GenePharma, China) according to the manufacturer’s instructions.

### Immunofluorescence (IF) assay

Glass slides with cell seedings were fixed in 4% paraformaldehyde, permeabilized in 0.2% TritonX-100, and then blocked with 5% BSA at room temperature. The primary antibodies were then applied to the cell slides and incubated overnight at 4 °C. Following PBST washes, the cell slides were incubated at room temperature and away from light with fluorescent-conjugated secondary antibodies, Goat Anti-Rabbit Alexa Fluor 488 or Goat Anti-Rabbit Alexa Fluor 647 (Beyotime, China). The nuclei were counterstained with DAPI for five minutes. The fluorescence was observed using Laser Scanning Confocal Microscope (Nikon C2, Japan).

### Cell transfection

Details of the cell transfection experiments were described in Supplementary File 1.

### RNA binding protein immunoprecipitation (RIP) assay

In brief, circHECTD1-Wt or circHECTD1-Mut plasmids with the putative RBMS3 binding site mutation were transfected into GBM cells. Magnetic bead-conjugated RBMS3 antibody (with IgG as a control) was obtained in cell lysates. After incubation, centrifugation, and rinsing, protein complexes were obtained. Proteinase K digestion and extraction of RNA using TRIzol reagent. The expression level of circHECTD1 was detected by qPCR.

### Co-immunoprecipitation (Co-IP) assay

Following the manufacturer’s instructions, Co-IP assays were carried out using the Pierce Co-IP Kit from Thermo Fisher, Waltham, Massachusetts. AminoLink Plus Coupling Resin immobilized primary antibody was produced, and cell lysates were then treated with them overnight at 4 °C. The samples were then eluted with Elution Buffer after being washed three times with Wash Buffer. The eluates were finally analyzed by western blot.

### Mass spectrometry (MS) analysis

Proteins were separated by SDS-PAGE gel electrophoresis, and the desired protein bands were removed from the gel. Following elution, reduction, and alkylation, the proteins were digested overnight at 37 °C. Collecting, desalting, and analyzing peptides with a timsTOF pro mass spectrometer (Bruker, Germany). Using NCBI’s nonredundant protein database with Mascot Daemon, sequence and site identification was examined.

### Cycloheximide (CHX) chase assay

The half-life of NR2F1 was determined using the protein synthesis inhibitor CHX. After being exposed to 100 mg/ml CHX (NobleRyder, China), GBM cells were harvested after 0, 4, 8, and 12 h. The expression levels of total proteins were then determined using a western blot.

### Immunoprecipitation and immunoblot analysis

Details of the immunoprecipitation and immunoblot analysis were described in Supplementary File 1.

### Chromatin immunoprecipitation (ChIP) assay

Using the SimpleChIP enzymatic ChIP kit (Cell Signaling Technology, USA), ChIP assay was performed. The following antibodies were used: anti-NR2F1 antibody (Catalog #sc-74560, Santa Cruz Biotechnology) and normal IgG. Anti-NR2F1 and anti-IgG immunoprecipitated DNA was amplified by PCR using primers. Supplementary Table 2 included the primers for each PCR set and the sizes of the PCR products. For validation of each PCR, the relevant input was obtained in parallel.

### Dual-luciferase reporter assay

The construction of reporter vectors was accomplished by introducing full-length, truncated, or mutant IRES into dual luciferase reporter vectors. 48 h after transfection, relative luciferase activities were measured using the Dual Luciferase® Reporter Assay System Kit (Promega, USA) in accordance with the manufacturer’s instructions.

### Cell counting Kit 8 assay

After performing cell suspension counts, 3000 GBM cells were resuspended in 100 μL DMEM containing 10% FBS, and seeded in 96-well plates. 10 μL CCK-8 was added and incubated for 2 h. To measure OD values, a plate reader with a wavelength of 450 nm was used. All experiments were repeated three times.

### Cell Migration, transwell and in vitro tube formation assay

The migration, invasion and VM channel formation capacity of GBM cells were determined by cell migration, transwell and in vitro tube formation assay. Details of the above assays were described in Supplementary File 1.

### Tumor xenografts in nude mice

All tests on nude mice were conducted under strict adherence to the protocol approved by the Administrative Panel on Laboratory Animal Care of China Medical University. Details were described in Supplementary File 1

### Statistical analysis

All experimental data were manifested as mean ± SD. For statistical analysis, GraphPad Prism 8 (GraphPad, USA) was used. Group comparisons were made with a one-way ANOVA or Student’s *t*-test. When *P* < 0.05, differences were deemed statistically significant.

## Results

### Identification and characteristics of circHECTD1 in GBM

**Fig. 1:**
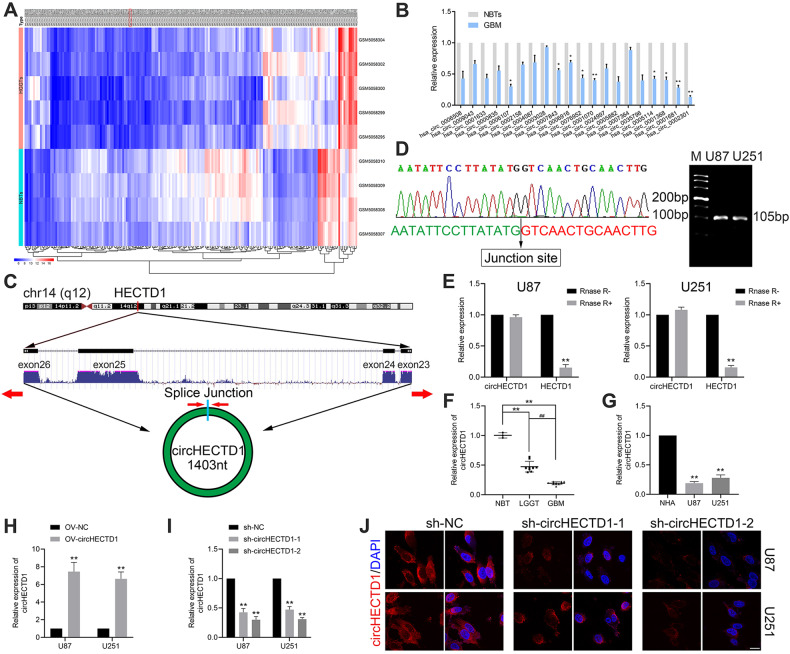
Characterization of the existence and subcellular distribution of circHECTD1 in GBM tissues and cells. **A** The cluster heat map from microarray data in GSE165926 shows circRNAs with different expression patterns among 9 tissue samples [NBT (GSM5058307, GSM5058308, GSM5058309, GSM5058310) and GBM tissues (GSM5058295, GSM5058299, GSM5058300, GSM5058302, GSM5058304)]. **B** Relative expression of top 20 differentially expressed consistent circRNAs (*n* = 3). *P < 0.05, **P < 0.01 vs NBT group. **C** Illustration of the annotated genomic region of HECTD1, the putative different mRNA splicing forms, and the validation strategy for the circular exon 23–26 (circHECTD1). **D** The circular junction of circHECTD1 was identified by using divergent primers on Sanger sequencing. **E** qPCR analysis of circHECTD1 and linear HECTD1 mRNA after treatment with RNase R in U87 and U251 cells (*n* = 3). **P < 0.01 vs circHECTD1 group. **F** Relative expression of circHECTD1 with qPCR analysis in NBT (*n* = 3), LGGT (*n* = 9), and GBM (*n* = 9). **P < 0.01 vs NBT group; ^##^*P* < 0.01 vs LGGT group. **G** Relative expression of circHECTD1 with qPCR analysis in NHA, U87, and U251 cells (*n* = 3). **P < 0.01 vs NHA group. **H**, **I** Transfection efficiency of circHECTD1 plasmids in GBM cells (*n* = 3). **P < 0.01 vs OV-NC group (left panel); **P < 0.01 vs sh-NC group (right panel). **J** The subcellular localization of circHECTD1 was shown by RNA FISH assay in sh-NC or circHECTD1 junction shRNAs-transfected GBM cells. Scale bar = 10 μm.

First, the expression of circRNAs in normal brain and GBM tissues were analyzed jointly using microarray datasets (GSE165926 and GSE92322) to identify circRNAs that may be differentially expressed (Fig. 1A, Supplementary Fig. 1A). The downregulated expressed circRNAs in the two datasets were analyzed, and the top 20 circRNAs with consistent expression changes were taken for further qPCR validation, and the lowest expressed hsa_circ_0002301 was found in GBM tissues (Fig. 1B). As shown in Fig. 1C, circHECTD1 was transcribed from exons 23-26 of HECTD1 on chr14:31596990-31602881 (14q12), ultimately forming a mature 1403nt sequence in length. Head-to-tail splicing of circHECTD1 was verified by Sanger sequencing of the circHECTD1 junction sequence, using specific primers and PCR assay, and the results were consistent with those of circBase (Fig. 1D). To further confirm the presence of circHECTD1, the resistance of circHECTD1 to RNase R treatment was assessed by applying an RNase R degradation assay. The linear transcripts of HECTD1 were degraded by RNase R treatment, whereas this treatment failed to degrade the circular transcripts of circHECTD1 (Fig. 1E). Further, we demonstrated that circHECTD1 expression decreased with increasing pathological grade of glioma (Fig. 1F). The expression of circHECTD1 was significantly downregulated in GBM cell lines (U87 and U251) compared to that in NHA (Fig. 1G). Overexpression by artificial plasmids induced a significant upregulation, and knockdown by two specific junction shRNAs caused a substantial downregulation of circHECTD1 expression level without affecting linear HECTD1 expression (Fig. 1H, I, Supplementary Fig. 1B). The RNA fluorescence in situ hybridization (FISH) assay indicated that circHECTD1 was located in both the nucleus and cytoplasm of GBM cells but mainly in the cytoplasm (Supplementary Fig. 1C). The circHECTD1 junction-specific RNA FISH probe further confirmed the efficiency and specificity of both shRNAs (Fig. 1J). Based on these results, circHECTD1 was selected as a potential functional circRNA in GBM.

### circHECTD1 acted as a crucial VM-suppressed factor in GBM cells

**Fig. 2:**
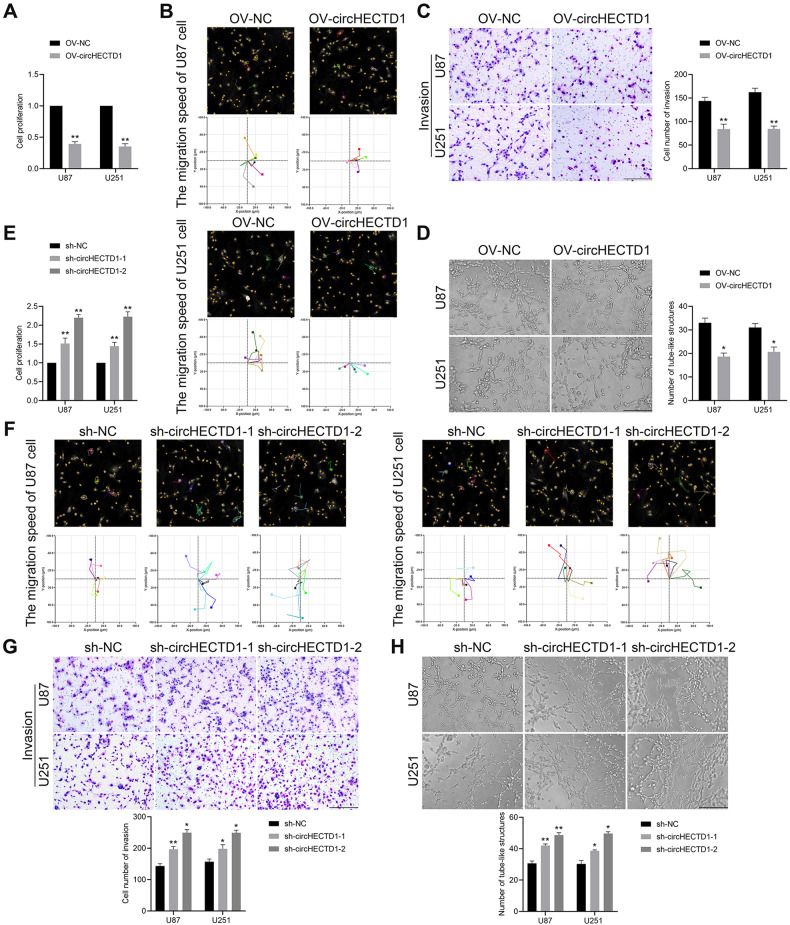
CircHECTD1 inhibited cell proliferation, migration, invasion, and VM channel formation in GBM cells. **A** CircHECTD1 elevation repressed the proliferation of U87 and U251 cells. **B** Overexpression of circHECTD1 inhibited the migration of U87 and U251 cells shown by digital holographic microscopy. **C** Overexpression of circHECTD1 inhibited the invasion of U87 and U251 cells shown by transwell assay. **D** circHECTD1 overexpression decreased the number of tube-like structures demonstrated by in vitro tube formation assay (*n* = 3). *P < 0.05, **P < 0.01 vs OV-NC group. **E** circHECTD1 reduction promoted the proliferation of GBM cells shown by CCK8 assay. **F** Knockdown of circHECTD1 promoted the migration of U87 and U251 cells. **G** circHECTD1 reduction enhanced cell invasive ability of U87 and U251 cells. **H** Knockdown of circHECTD1 promoted the tube formation (*n* = 3). *P < 0.05, **P < 0.01 vs sh-NC group. Scale bar = 200 μm.

To clarify whether circHECTD1 is involved in regulating VM formation in GBM, gain- and loss-of-function approaches were utilized. CCK8 assay indicated that circHECTD1 overexpression inhibited the proliferation of U87 and U251 cells (Fig. 2A). Subsequently, we explored the impact of circHECTD1 on GBM cell motility. circHECTD1 overexpression also impeded U87 and U251 cells from migrating and invading (Fig. 2B, C). The in vitro tube formation assay indicated that the upregulation of circHECTD1 induced an obvious decrease in the number of tubular structures in both cell lines (Fig. 2D). Immediately after, two specific shRNAs were used to knock down circHECTD1 expression to evaluate its VM-related function in GBM cells. circHECTD1 reduction significantly promoted cell proliferation, migration, invasion, and in vitro tube formation in GBM cells (Fig. 2E–H), and the promotion of the above VM formation effect by sh-circHECTD1-2 was more pronounced. Further, the expression of proteins related to VM was tested. Elevated circHECTD1 markedly reduced MMP2, MMP9, and VE-cadherin protein levels and vice versa (Supplementary Fig. 1D, E). Collectively, these results suggest that circHECTD1 is a key regulator of VM formation in GBM.

### RBMS3 inhibited proliferation, migration, invasion, and tubule formation in GBM cells

Further, the study intended to investigate the formation mechanism of circHECTD1. The bioinformatics website MEME Suite was first used to analyze the flanking intron sequence 500 bp upstream and downstream of the circHECTD1 splice site. And the flanking intron sequence of circHECTD1 was found to be enriched with multiple RBP motifs (Supplementary Fig. 1F). Numerous studies have shown that RBPs play an important regulatory role in the formation of circRNAs [28]. Subsequently, RBPmap combined with MEME Suite were applied to predict RBPs that bind to the flanking intron sequences at both ends. As a result, 14 candidate RBPs are obtained by taking the intersection (Supplementary Fig. 1G). Further analysis of the TCGA and GEPIA database revealed that among the 14 candidates, only the low expression of RBMS3 and CPEB4 were positively correlated with poor prognosis in glioma patients (Supplementary Fig. 1H, I). qPCR assay was utilized and the results showed that RBMS3 inhibition significantly decreased the expression of circHECTD1, while knockdown of CPEB4 had no effect on circHECTD1 expression (Supplementary Fig. 1J). Therefore, RBMS3 was chosen to investigate its regulatory mechanism with circHECTD1.

**Fig. 3:**
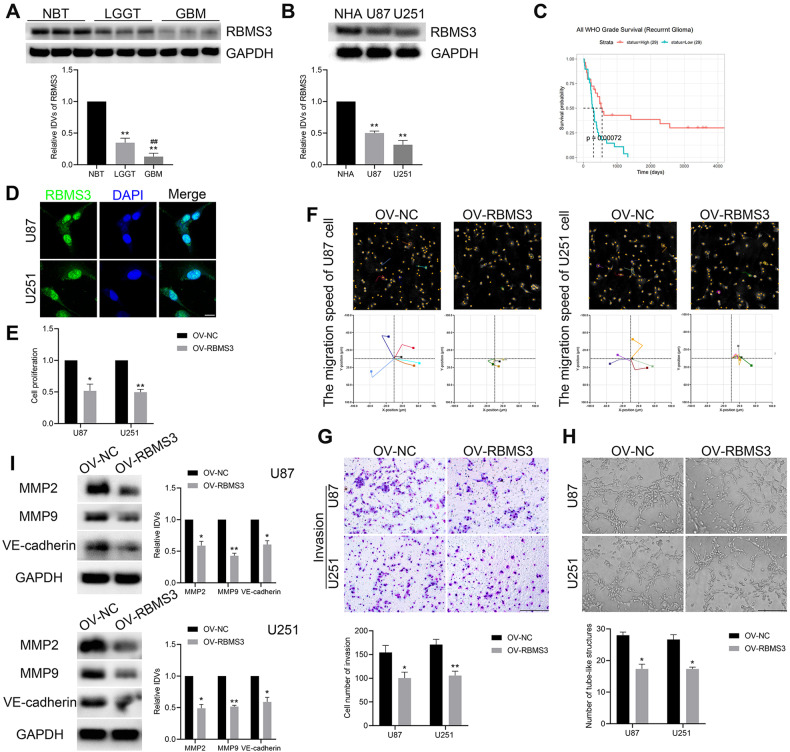
RBMS3 depressed the VM formation of GBM cells. **A** Relative expression of RBMS3 in NBT (*n* = 3), LGGT (*n* = 9), and GBM (*n* = 9) shown by western blot. **P < 0.01 vs NBT group; ^##^*P* < 0.01 vs LGGT group. **B** The endogenous expression of RBMS3 in NHA, U87, and U251 cells (*n* = 3). **P < 0.01 vs NHA group. **C** Effect of RBMS3 expression level on recurrent glioma patient survival time from CGGA database. **D** The subcellular localization of RBMS3 in U87 and U251 cells was shown by IF assay. Scale bar = 10 μm. **E** RBMS3 inhibited U87 and U251 cell proliferation shown by CCK8 assay. **F**, **G** RBMS3 suppressed the migration and invasion capacity of U87 and U251 cells. **H** RBMS3 decreased the number of VM-channel of U87 and U251 cells shown by in vitro tube formation assay. Scale bar = 200 μm. **I** Overexpression of RBMS3 reduced the expression level of MMP2, MMP9, and VE-cadherin shown by western blot (*n* = 3). *P < 0.05, **P < 0.01 vs OV-NC group.

The study demonstrated that RBMS3 expression was significantly and consistently reduced in glioma tissues compared to that in NBTs (Fig. 3A). Moreover, the expression of RBMS3 was markedly downregulated in GBM cells compared to that in NHA (Fig. 3B). Kaplan-Meier curve survival analysis demonstrated a worse overall survival in patients with lower expression of RBMS3, which is relevant to recurrent glioma (Fig. 3C, Supplementary Fig. 1I). The IF assay showed that RBMS3 was mainly localized in the nucleus in GBM cells (Fig. 3D). Afterward, an RBMS3 overexpression plasmid was constructed and used to probe the VM function of RBMS3 in GBM cells. As expected, analysis using the CCK8 assay indicated that the proliferative ability of GBM cells was significantly weakened upon RBMS3 overexpression (Fig. 3E). Digital holographic microscopy and transwell assays demonstrated that RBMS3 elevation prominently attenuated the cell migration and invasion ability (Fig. 3F–G). A subsequent in vitro tube formation assay was used to assess the vascular-like tubes formation on Matrigel. As shown in Fig. 3H, RBMS3 overexpression inhibited VM in GBM cells in vitro. Additionally, increased RBMS3 expression resulted in significantly reduced expression of VM-related proteins (Fig. 3I). Taken together, our results demonstrated that RBMS3 overexpression restrained VM in GBM cells.

### RBMS3 promoted circHECTD1 generation in GBM cells

**Fig. 4:**
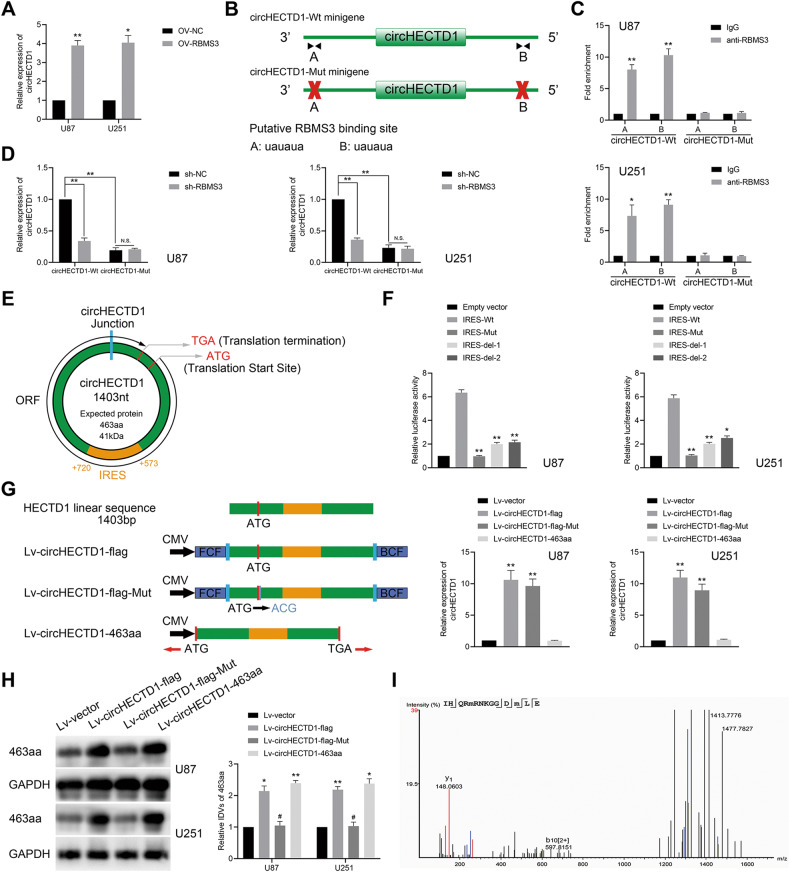
RBMS3 bound to and promoted the production of circHECTD1, which had an encoding function. **A** Relative expression of circHECTD1 in U87 and U251 cells with RBMS3 overexpression was shown by qPCR assay (*n* = 3). *P < 0.05 vs OV-NC group; **P < 0.01 vs OV-NC group. **B** The illustration of the putative RBMS3-binding sites on the flanking introns in the circHECTD1-Wt and circHECTD1-Mut minigenes. **C** RIP analysis of RBMS3 binding to circHECTD1-Wt and circHECTD1-Mut minigenes in U87 and U251 cells (*n* = 3). *P < 0.05 vs IgG group; **P < 0.01 vs IgG group. **D** qPCR analysis of circHECTD1 expression in U87 and U251 cells co-transfected with sh-RBMS3 and circHECTD1 minigenes (*n* = 3). **P < 0.01 vs sh-NC group. **E** The illustration of putative ORF and IRES in circHECTD1. **F** IRES sequences in circHECTD1 or its different truncations or mutation were cloned between Rluc and Luc reporter genes with independent start and stop codons. The relative luciferase activity of Luc/Rluc in the above vectors was tested (*n* = 3). *P < 0.05, **P < 0.01 vs IRES-Wt group. **G** Four vectors were constructed. Lv-vector; Lv-circHECTD1-flag: flag-labeled circHECTD1 sequence was cloned into a CMV-induced expression vector; Lv-circHECTD1-flag-Mut: flag-labeled circ circHECTD1 sequence with start codon mutant (ATG → ACG) was cloned into a CMV-induced expression vector; Lv-circHECTD1-463aa: flag-labeled circHECTD1-463aa sequence was cloned into a CMV-induced expression vector. Relative expression of circHECTD1 was tested by qPCR assay (*n* = 3). **P < 0.01 vs Lv-vector group. **H** The expression level of 463aa was shown by western blot (*n* = 3). *P < 0.05, **P < 0.01 vs Lv-vector group; ^#^*P* < 0.05 vs Lv-circHECTD1-flag group. **I** circHECTD1-463aa junction-specific peptide was identified.

The potential binding sites between RBMS3 and circHECTD1 flanking intron sequences were further analyzed through RBPmap (Supplementary Fig. 1K). This study found that RBMS3 upregulation led to a significant increase in circHECTD1 expression in GBM cells, while linear HECTD1 expression remained unchanged (Fig. 4A, Supplementary Fig. 1L). Further, two short circHECTD1 minigenes (circHECTD1-Wt, circHECTD1-Mut) were generated to explore their potential mechanisms. The circHECTD1-Wt minigene contained putative RBMS3-binding sites on both flanking introns, which were retained, whereas the binding sites on the circHECTD1-Mut minigene were deleted (Fig. 4B). RIP analysis demonstrated a clear interaction between RBMS3 and circHECTD1-Wt, but not circHECTD1-Mut (Fig. 4C), suggesting that RBMS3 bound to the putative binding sites on the flanking introns. Next, it has been found that the circHECTD1-Mut minigene produced significantly less circHECTD1 than circHECTD1-Wt, and circHECTD1-Wt produced lower levels of circHECTD1 transcript with a lack of RBMS3 (Fig. 4D).

Our study further investigated whether RBMS3 promoted the production of circHECTD1 to affect VM formation in GBM. The results showed that circHECTD1 overexpression significantly exacerbated the inhibition of cell proliferation, migration, invasion, and tubule formation caused by RBMS3 elevation. And compared with RBMS3 inhibition, knockdown of circHECTD1 (using sh-circHECTD1-2 plasmid with higher transfection efficiency) effectively increased the VM formation capacity of GBM cells (Supplementary Fig. 2). These results confirm that RBMS3 effectively promoted circHECTD1 production to inhibit VM formation in GBM cells.

### circHECTD1 encoded a novel 463 amino acid peptide termed 463aa

circHECTD1 has been reported to act as a miRNA sponge to regulate tumor progression [29]. However, regulatory role of circHECTD1 in VM formation is still unknown. To investigate whether circHECTD1 acts as a sponge for miRNAs to regulate VM formation in GBM, AGO2, a key factor in miRNA-mediated gene silencing, was first proposed to be tested. Through bioinformatics analysis of CircInteractome, we did not find the interaction possibility between circHECTD1 and AGO2 (Supplementary Fig. 3A). Further, RIP assays were used to detect whether circHECTD1 bound to AGO2, and we observed no circHECTD1 enrichment in AGO2 precipitates (Supplementary Fig. 3B). Therefore, we assumed that circHECTD1 might not work as miRNAs sponge in GBM.

Subsequently, we explored the mechanisms by which circHECTD1 inhibited VM formation in GBM. By searching the circRNADb database, we found that circHECTD1 contains an ORF and IRES sequences with a large protein-coding potential (Fig. 4E, Supplementary Fig. 3C). Conservation analysis showed that 463aa encoded by this ORF is highly conserved among different species, suggesting that it is translatable (Supplementary Fig. 3D). Recent studies have shown that the putative IRES sequences are also required for non-coding RNAs to perform 5’ cap-independent coding functions [30]. Truncated or full-length putative circHECTD1 IRES sequence were cloned between the Rluc and Luc reporter genes to test IRES activity in circHECTD1. The dual luciferase reporter assay demonstrated that IRES-Wt induced the highest luciferase activity compared to the IRES-Mut and IRES-del groups (Fig. 4F, Supplementary Fig. 3E). These results indicated that the putative IRES sequence in circHECTD1 may be highly competent at initiating protein translation.

To dissociate the role of 463aa from circHECTD1, several flag-labeled vectors for circHECTD1 were constructed and a 463aa-specific antibody was designed and synthesized. The expression level of circHECTD1 was significantly elevated in GBM cells transfected with Lv-circHECTD1-flag and Lv-circHECTD1-flag-Mut vectors, whereas neither the control vector nor Lv-circHECTD1-463aa produced similar effects (Fig. 4G). And transfection with Lv-circHECTD1-flag and Lv-circHECTD1-463aa vectors both successfully led to 463aa elevation, while the control vector or Lv-circHECTD1-flag-Mut did not (Fig. 4H). Moreover, circHECTD1 knockdown markedly reduced the expression level of 463aa (Supplementary Fig. 3F). The translation of 463aa from circHECTD1 was further confirmed by LC-MS/MS, while the unique amino acid sequence of 463aa (IHQRMRNKGGDM) was determined (Fig. 4I). Taken together, circHECTD1 had a novel function of encoding protein and encoded a conserved 463aa.

### circHECTD1 and 463aa inhibited the GBM VM formation in vitro and in vivo

**Fig. 5:**
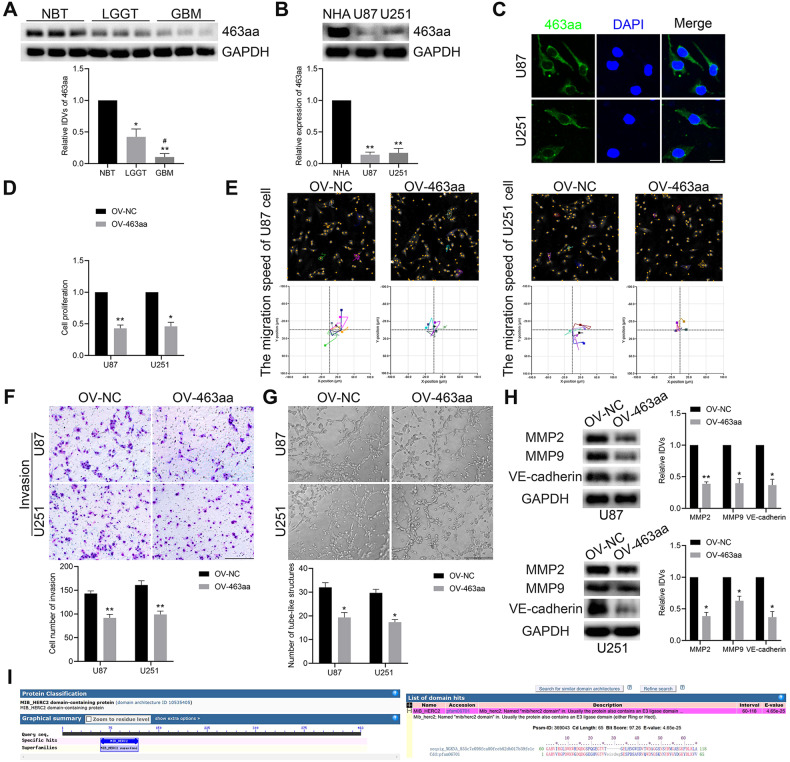
CircHECTD1-463aa inhibited the proliferation, migration, invasion, and VM formation in GBM cells. **A** Relative expression of 463aa in NBT (*n* = 3), LGGT (*n* = 9), and GBM (*n* = 9) was shown by western blot; *P < 0.05, **P < 0.01 vs NBT group; ^##^*P* < 0.01 vs LGGT group. **B** The expression level of 463aa in NHA, U87, and U251 cells (*n* = 3). **P < 0.01 vs NHA group. **C** Subcellular distribution of 463aa in U87 and U251 cells shown by IF assay. Scale bar = 10 μm. **D**–**G** 463aa inhibited cell proliferation, migration, invasion, and VM-channel formation of U87 and U251 cells. Scale bar = 200 μm. **H** Overexpression of 463aa decreased the expression level of VM-related protein shown by western blot (*n* = 3). *P < 0.05, **P < 0.01 vs OV-NC group. **I** The sequence analysis of the 463aa domain from the NCBI database.

Further 463aa effects on VM in GBM are presented for determination. As shown in Fig. 5A, 463aa was markedly downregulated in LGGT and GBM compared with NBT, while its expression decreased with increasing pathological grade. Similar results were obtained in further validation using IHC assays in GBM tissues (Supplementary Fig. 3G). And NHA displayed higher levels of 463aa expression than GBM cells (Fig. 5B). IF staining showed that 463aa was mainly distributed in the cytoplasm of GBM cells (Fig. 5C). Overexpression of 463aa resulted in decreased proliferation, as evidenced by the CCK8 assay in GBM cells (Fig. 5D). For the motility of GBM cells, 463aa upregulation also inhibited the migration and invasion abilities (Fig. 5E, F). GBM cells with 463aa elevation showed less tubular-type VM channel formation and lower expression levels of VM-related proteins (Fig. 5G, H). In addition, upregulation of 463aa reversed the promotion of VM formation in GBM cells by circHECTD1 knockdown (Supplementary Fig. 4). Collectively, 463aa had the ability to inhibit VM formation in GBM.

**Fig. 6:**
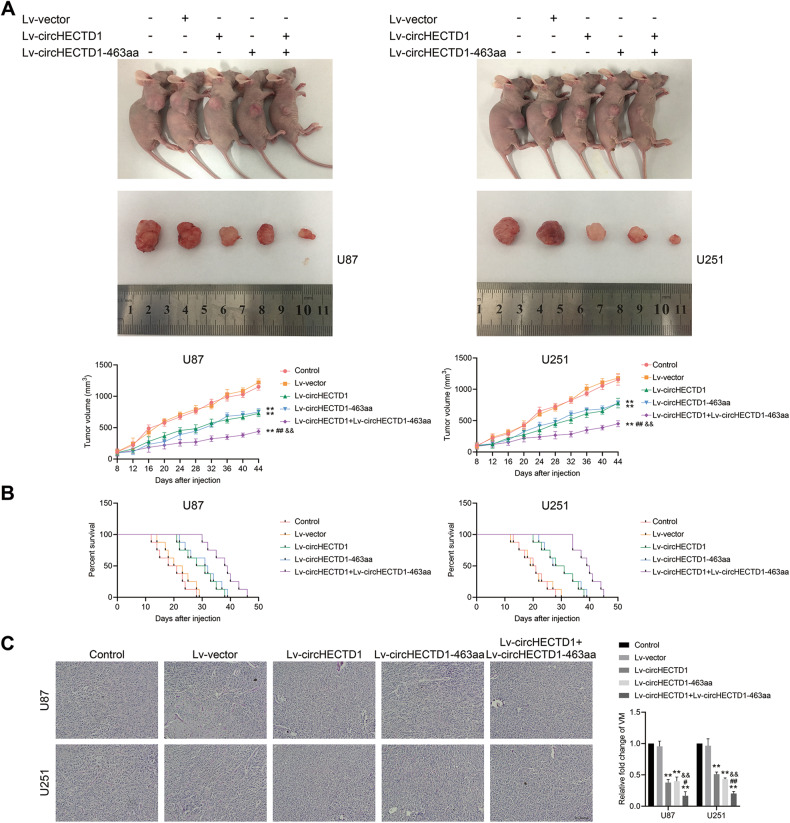
CircHECTD1 and 463aa inhibited tumor growth and VM formation in vivo. **A** Subcutaneously implanted tumor model in nude mice was constructed by U87 and U251 cells transfected with Lv-circHECTD1 and Lv-circHECTD1-463aa vectors. Tumor volume was recorded every 4 days, and tumor growth curves were plotted. **P < 0.01 vs Lv-vector group; ^##^*P* < 0.01 vs Lv-circHECTD1 group; ^&&^*P* < 0.01 vs Lv-circHECTD1-463aa group. **B** Orthotopic transplantation tumor model in nude mice was generated by the above vectors. Survival curves from representative nude mice injected into the right striatum were shown (*n* = 8). **C** PAS/CD31 dual-staining assay was used to detect the effects of the above vectors on VM in orthotopically transplanted tumor tissue of nude mice (*n* = 3). **P < 0.01 vs Lv-vector group; ^#^*P* < 0.05, ^##^*P* < 0.01 vs Lv-circHECTD1 group; ^&&^*P* < 0.01 vs Lv-circHECTD1-463aa group. Scale bar = 200 μm.

A nude mice GBM xenograft assay was performed to test the effect of circHECTD1 and 463aa in regulating VM tube formation in vivo. As shown in Fig. 6A, circHECTD1 and 463aa overexpression individually produced smaller tumors compared to the negative control group. However, GBM cells treated with dual-overexpressing circHECTD1 and 463aa generated the smallest tumor volumes among those groups. Additionally, the upregulation of circHECTD1 and 463aa significantly prolonged the survival time of orthotopic tumor-bearing mice compared to the negative control group, and the combination of these two showed a more substantial survival time prolongation effect (Fig. 6B). Furthermore, PAS/CD31 dual staining assay was used to verify the effect of the above treatment on the number of VM channels in orthotopically transplanted tumor tissue. VM is characterized by PAS-positive staining and CD31-negative staining [1]. As shown in Fig. 6C, there were fewer VM channels in mice-transplanted tumors with circHECTD1 and 463aa elevation, and VM channels were reduced the most in transplanted tumors with circHECTD1 and 463aa dual-overexpression. Taken together, circHECTD1 and the encoded-463aa inhibiting GBM VM formation in vitro and in vivo.

### 463aa positively regulated NR2F1 ubiquitination

**Fig. 7:**
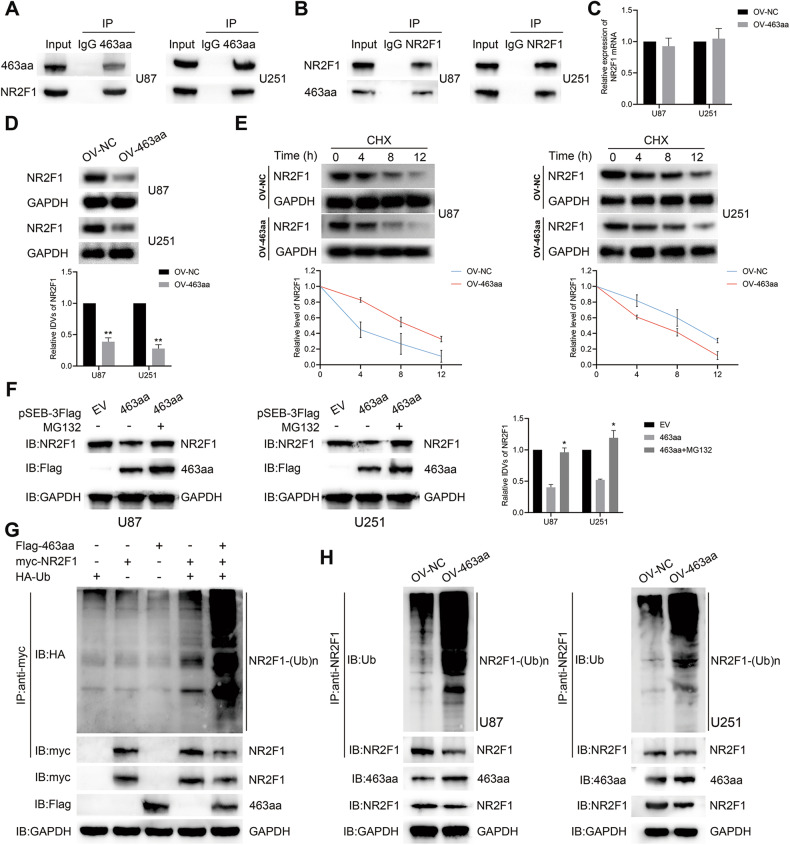
463aa modulated the ubiquitination modification of NR2F1. **A**, **B** 463aa and NR2F1 complexes were co-immunoprecipitated with anti-463aa and anti-NR2F1 antibodies, and NR2F1 and 463aa were detected, respectively. **C** Relative expression of NR2F1 mRNA in 463aa overexpression GBM cells was tested by qPCR assay. **D** Relative expression of NR2F1 protein in U87 and U251 cells with 463aa overexpression was tested by western blot (*n* = 3). **P < 0.01 vs OV-NC group. **E** U87 and U251 cells with 463aa elevation were incubated with CHX for the indicated times. The relative expression of NR2F1 protein level was shown. **F** Control or 463aa-overexpressing GBM cells were treated with CHX for the indicated time, then were treated with or without the proteasome inhibitor MG132 for 2 h before collection. The expression of 463aa and NR2F1 were determined and quantified (*n* = 3). *P < 0.05 vs 463aa group. **G** HA-Ubiquitin, myc-NR2F1, or Flag-463aa plasmids were transfected alone or together into HEK293T cells as indicated. NR2F1 ubiquitination was detected by immunoprecipitation of 463aa with anti-myc antibody and western blot with anti-HA antibody. The expression of NR2F1 and 463aa in the whole-cell lysates was confirmed. **H** The ubiquitination of endogenous NR2F1 was analyzed by immunoprecipitation in U87 and U251 cells overexpressing 463aa.

A bioinformatics database was used to analyze the 463aa domain to investigate the molecular mechanism by which 463aa is involved. We found that 463aa contained the Mib_HERC2 domain (Fig. 5I). According to previous studies, several proteins containing this domain are involved in the ubiquitination modification progress [31, 32], and the application of InterPro database analysis found that 463aa may possess the molecular function of ubiquitin-protein transferase activity (Supplementary Fig. 5A), so we reasonably doubt whether 463aa gets involved. To test this hypothesis, we first identified the proteins that interacted with 463aa by performing IP combined with MS analyses, and screened the NR2F1 (Supplementary Fig. 5B). NR2F1 is a transcription factor whose functions remain unknown in GBM. We further confirmed that NR2F1 was present in 463aa complexes, and 463aa was confirmed to exist in NR2F1 complexes (Fig. 7A, B), indicating that NR2F1 and 463aa could interact with each other in GBM cells. Moreover, overexpression of 463aa did not alter the mRNA expression of NR2F1, whereas markedly decreased NR2F1 protein levels (Fig. 7C, D). The IF assay further confirmed that 463aa and NR2F1 were mainly co-localized in the cytoplasm of U87 and U251 cells (Supplementary Fig. 5C). Hence, we speculated that 463aa mediates the degradation of NR2F1 rather than altering its transcription. Cycloheximide (CHX) was used to measure the half-life of NR2F1 protein and showed that 463aa upregulation shortened the half-life (Fig. 7E), which further validated the hypothesis. MG132, a widely used proteasome inhibitor, rescued the degradation of NR2F1 induced by 463aa overexpression (Fig. 7F), confirming that 463aa may regulate the expression level of NR2F1 protein through the proteasome pathway. Furthermore, the co-expression of 463aa with NR2F1 significantly increased its ubiquitination (Fig. 7G). The endogenous ubiquitination of NR2F1 was examined in GBM cells, which showed that 463aa overexpression led to elevated NR2F1 ubiquitination compared to the NC group (Fig. 7H). To map the NR2F1 ubiquitination sites, mutagenesis was used to inactivate the putative residues according to the Uniprot database. K396 mutation was found to decrease the 463aa-mediated polyubiquitination of NR2F1 (Supplementary Fig. 5D, E). Collectively, these results suggested that 463aa positively regulated NR2F1 ubiquitination in GBM cells.

### NR2F1 transcriptionally activated VM-related proteins expression in GBM cells

The endogenous expression of NR2F1 in glioma tissues and cells was first determined. We found that NR2F1 was markedly increased in glioma tissues and cells compared to NBT and NHA (Supplementary Fig. 6A, B). The IF assay demonstrated its localization in GBM cells (Supplementary Fig. 6C). To investigate whether NR2F1 participated in the regulation of VM in GBM cells, we transfected GBM cells with an NR2F1 overexpression plasmid and knocked down NR2F1 with shRNA. The results showed that enforced NR2F1 expression markedly enhanced cell proliferation, migration, invasion, and tube-forming capacity. However, NR2F1 knockdown significantly suppressed VM in GBM cells (Supplementary Fig. 6D–G). This implied that NR2F1, which was highly expressed in GBM cells, promoted VM formation. Further, we found that overexpression of NR2F1 significantly recovered the inhibiton of VM formation in GBM cells caused by 463aa elevation (Supplementary Fig. 7).

**Fig. 8:**
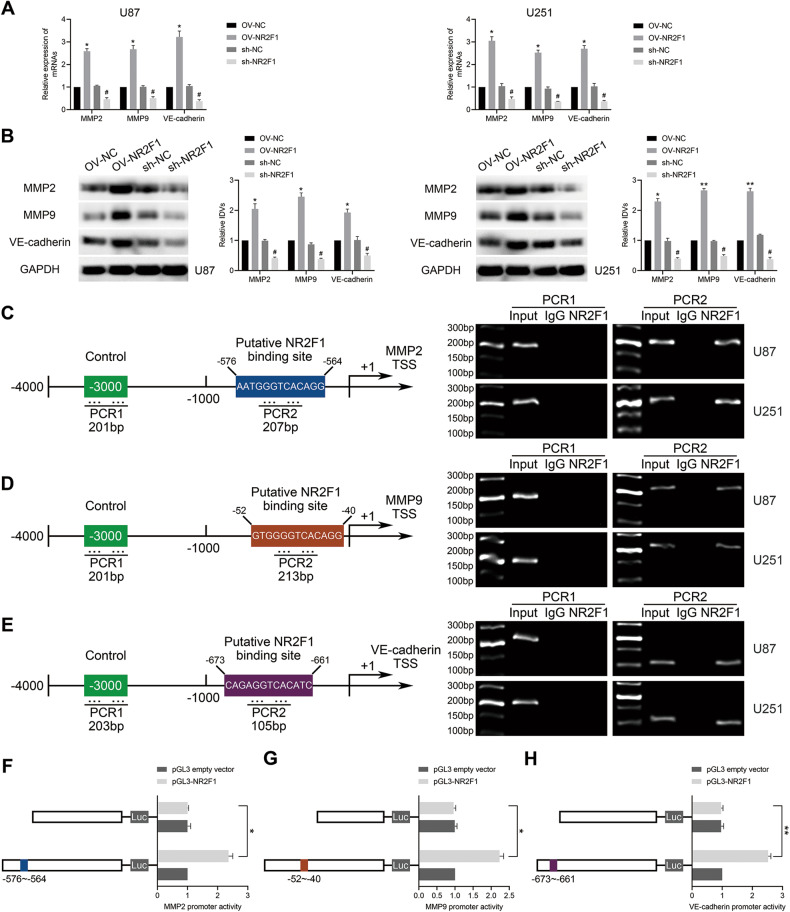
NR2F1 bound to the promoter region of MMP2, MMP9, and VE-cadherin. **A** Relative mRNA expression of MMP2, MMP9, and VE-cadherin was tested in U87 and U251 cells with overexpressing or knockdown NR2F1 by qPCR assay (*n* = 3). *P < 0.05 vs OV-NC group; ^#^*P* < 0.05 vs sh-NC group. **B** Relative expression of VM-related protein was shown (*n* = 3). *P < 0.05, **P < 0.01 vs OV-NC group; ^#^*P* < 0.05 vs sh-NC group. **C**–**E** Schematic representation of the MMP2, MMP9, and VE-cadherin promoter region 1000 bp upstream of TSS, which was designated as +1, was shown. The putative NR2F1 binding site was indicated. PCR was conducted with the resulting precipitated DNA. **F**–**H** Schematic depiction of the different reporter plasmids and relative luciferase activity. The relative luciferase activity was performed after cells were co-transfected with the promoter (−1000 to 0 bp) (or promoter-deleted putative NR2F1 binding site) with pGL3 empty promoter or pGL3-NR2F1 (*n* = 3). *P < 0.05, **P < 0.01 vs pGL3-NR2F1 group.

Given the involvement of NR2F1 in the modulation of transcriptional processes during tumor cell dormancy [33, 34], we further explored its transcriptional regulatory role in GBM cells. JASPAR database was used to identify the putative binding sites for NR2F1 in the region 2 kb upstream of MMP2, MMP9, and VE-cadherin transcription start sites (Supplementary Fig. 6H–J). Therefore, we first applied dysregulation of NR2F1 to observe its effect on the mRNA and protein expression of these factors. Upregulation of NR2F1 markedly stimulated the mRNA and protein expression of VM-related proteins, whereas shRNA-mediated knockdown of NR2F1 reduced the expression level (Fig. 8A, B). Furthermore, ChIP analysis demonstrated that NR2F1 could directly bind to the MMP2, MMP9, and VE-cadherin promoters in GBM cells (Fig. 8C–E). The luciferase reporter assay revealed that NR2F1 overexpression notably elevated the promoter-driven luciferase activities of VM-related proteins, while truncated gene promoter sequences with predicted binding sites dramatically displayed decreased promoter activity (Fig. 8F–H). In conclusion, NR2F1 transcriptionally activated the expression of MMP2, MMP9, and VE-cadherin in GBM cells.

## Discussion

This study demonstrated for the first time that RBMS3, which was downregulated in GBM tissues and cells, promoted the generation of circHECTD1 by binding to its flanking intron sequence. circHECTD1 owned a novel coding function and encoded a new functional peptide, 463aa, to play its key role in regulating VM formation. Analysis revealed that 463aa contained a ubiquitin transferase-related domain and experimentally verified its potential function in regulating NR2F1 ubiquitination. NR2F1 was a new ubiquitination substrate for 463aa, and mutating NR2F1-K396 significantly reduced the level of 463aa-mediated ubiquitination and increased its expression. Finally, NR2F1 was able to directly bind the promoter region of MMP2, MMP9 and VE-cadherin, transcriptionally promoting the expression of VM-associated proteins, which in turn promoted VM formation in GBM.

Here we report the mechanism of VM formation in GBM, modulated by circHECTD1. In the present study, through joint analysis of two GEO databases (GSE165926 and GSE92322), circHECTD1 with the lowest expression in several GBM tissues was screened for in-depth research. Further experimental verification revealed that circHECTD1 was significantly downregulated in GBM tissues and cells. CircHECTD1 elevation significantly repressed the proliferation, migration, invasion, and tube-forming capacities of GBM cells, which inhibited VM formation, suggesting that circHECTD1 plays a crucial role in GBM functioning as a VM suppressor. CircRNAs have attracted wide attention because of their unique structural characteristics and broad expression patterns in carcinogenesis [35]. The elevated circMMP9 acts as a molecular sponge for miR-124 and facilitates the GBM cell proliferation, migration, and invasion [36]. Moreover, the downregulation of circRNA-CDR1as inhibits p53/MDM2 complex formation by directly interacting with p53, thereby inhibiting GBM tumor growth [37].

Previous studies have indicated that circHECTD1 serves a critical regulatory function in the nervous system. CircHECTD1 knockdown reduced the TRAF3 expression by targeting miR-133b, thereby lightening neuronal damage caused by cerebral ischemia [38]. In addition, circHECTD1 suppression can also significantly reduce cerebral infarct size in mice with transient middle cerebral artery occlusion through the MIR142/TIPARP pathway, reduce neuronal damage, and promote the activation of astrocytes [39]. These results suggest that circHECTD1 may be an essential biomarker for CNS diseases such as ischemic stroke. In previous studies, circHECTD1 has been reported to regulate tumor progression, including cell proliferation and migration in glioma. Knockdown of hsa_circ_0000375 suppressed the proliferation of both LN229 and T98G cell lines by regulating the miR-296-3p/SLC10A7 axis [40]. CircHECTD1 targeting the miR-320-5p axis affects the proliferation and migration of C6 and U87 cells [41]. However, our study focused on an unresearched HECTD1 transcriptional variant, hsa_circ_0002301, and its function in GBM VM formation remains unknown.

Subsequently, this study focused on the regulation of circHECTD1 formation by RBMS3. We confirmed that suppression of RBMS3 increased the number of proliferating, migrating, and invasive GBM cells while at the same time facilitating GBM cell tube formation. RBMS3, an RNA-binding protein, participates in various processes, such as embryogenesis, cancer initiation and progression [42]. Low expression of RBMS3 in breast cancer tissue is significantly negatively correlated with poor prognosis [16]. RBMS3 inhibits breast cancer metastasis by regulating Twist1 expression [43]. In nasopharyngeal carcinoma, RBMS3 activates caspase-9 and PARP to promote tumor cell apoptosis in a mitochondria-dependent manner, repressing the microvessels formation via downregulating MMP2 and β-catenin [44]. Similarly, RBMS3 inhibits the β-catenin/CBP signaling pathway by competitively binding to miR-126-5p, thereby reducing chemotherapy resistance in epithelial ovarian cancer cells [45].

Furthermore, through analysis from the bioinformatics database, it was found that the flanking intron sequence of circHECTD1 contained the potential binding site of RBMS3. Therefore, two circHECTD1 minigenes with flanking sequences containing RBMS3 binding sites (UAUAUA) and binding-site mutations were constructed. RIP assays were used to confirm the direct binding between RBMS3 and circHECTD1 flanking intron sequences. Subsequently, it was verified that RBMS3 made a great difference in inhibiting GBM cell VM formation by modulating the formation of circHECTD1. According to previous reports, exonic circRNAs are formed by either lariat-driven, intron pairing-driven, or RBP-driven circularization [46]. Among these, RBP-driven circularization has always been a research hotspot. Previous studies have shown that RBPs participate in the regulation of circRNA formation by binding to particular motifs in flanking intron sequences [47]. For instance, RBM3 regulated the formation of SCD-circRNA 2 by interacting with the flanking sequence of the back-splicing site [48]. In human embryonic stem cells, RBP-ESRP1 promotes the circularization of circBIRC6 by binding to the ESRP1 binding motif in the flanking intron sequence, which in turn participates in regulating cell pluripotency and differentiation [49].

In this study, based on the circRNADb analysis, it was found that circHECTD1 has a length of 1392nt ORF and an active IRES, which may have the potential to encode a peptide. Furthermore, the encoding function of circHECD1 was clarified by customizing specific antibodies combining LC-MS/MS analysis to identify circHECTD1-encoded 463aa. To explore whether circHECD1-463aa is also involved in the regulation of VM in GBM cells, we examined its effect. It was found that 463aa, under-expressed in GBM cells and tissues, inhibited VM formation in GBM cells. Moreover, nude mice xenograft tumor assays proved that circHECTD1 and 463aa elevation, separately or simultaneously, can significantly reduce the tumor volume and prolong the survival period of nude mice. The combined application of the above treatments led to the smallest tumor volume and longest survival time. The PAS/CD31 dual-staining assay also showed a similar inhibitory effect on the number of VM channels. Taken together, circHECTD1 encoded 463aa repressing GBM VM formation in vivo and in vitro.

Emerging evidence shows that a few novel proteins are encoded by certain circRNAs in the regulation of glioma tumorigenesis [50, 51]. However, the involvement of protein-coding circRNAs in GBM VM remains unknown. Non-coding RNAs, such as circRNAs and lncRNAs, can be translated by ribosomes and perform coding functions [52, 53]. Studies have shown that partial circRNAs contain highly conserved ORFs, which are capable of encoding peptides in vitro and in vivo in a manner independent of the 5’cap structure, such as IRES induction, m^6^A methylation, and rolling cycle amplification [54]. Owing to the unique covalent closed-loop structures of circRNAs, ORFs involved in encoding can span the junction sites of circRNAs, and longer ORFs are more likely to function as encoded peptides [52]. For example, highly expressed circβ-catenin encodes the β-catenin-370aa, consisting of 370 amino acids, promoting the growth and metastasis of liver cancer via the Wnt/β-catenin signaling pathway [55]. A novel functional peptide 146aa is generated by circ-SHPRH in GBM cells via the overlapping start and stop codons “UGAUGA” produced as a result of cyclization, which plays a vital role in inhibiting GBM cell proliferation and tumorigenesis in vitro [56]. IRES is a type of RNA regulatory element that realizes ribosome assembly and translation of reading frame proteins by recruiting ribosomes, and initiates the process of direct translation of proteins. Early studies found that circRNAs constructed using IRES in vitro can recruit ribosomes and be translated to proteins [57]. At present, increasing evidence also shows that endogenous circRNAs with IRES can translate longer peptide chains through continuous ORFs and then participate in the regulation of cell differentiation, proliferation, stress response, and cell apoptosis [13].

To elucidate the 463aa function, we analyzed the sequence and found that it contains the MIB_HERC2 domain and may possess the molecular function of ubiquitin-protein transferase activity, so it is speculated that 463aa may be involved in regulating the ubiquitination of proteins in GBM cells. Ubiquitination is a widely studied post-translational modification pattern that refers to the covalent binding of ubiquitin to protein substrates and shows a great influence on tumorigenesis and development [58]. The circNEIL3 stabilizes IGF2BP3 via blocking HECTD4-mediated ubiquitination, thereby promoting the oncogenic progression of glioma [59]. In this study, IP combined with MS analysis was used to test the interaction between NR2F1 and 463aa in GBM cells. The co-expression of 463aa with NR2F1 significantly elevated the ubiquitination level of NR2F1. Moreover, the K396 mutation decreased 463aa-mediated polyubiquitination of 463aa. Collectively, these results confirmed that 463aa regulates the ubiquitination modification of NR2F1-K396 and inhibits VM formation in GBM cells. It is reported that circRNAs-encoded functional peptides may have the enzymatic activity to regulate the post-translational modification process of target genes. NTRK2-243aa-encoded by circNTRK2-phosphorylated PAX5 at Y102 regulated GBM glycolysis [60].

The study further found that NR2F1 was elevated in GBM cells and tissues, while its inhibition markedly decreased the number of proliferated, migrated, and invaded GBM cells as well as tubular-type VM channels. Transcription factor NR2F1 binds to the CXCL12 and CXCR4 promoter regions directly to promote their transcriptional expression, thus facilitating the metastasis and invasion of salivary adenoid cystic carcinoma cells [23]. Given the discovery of NR2F1 binding sites with the JASPAR database, a ChIP assay was used to verify that NR2F1 binds to the promoter region of VM-related proteins through these sites. NR2F1 knockdown significantly downregulated the mRNA and protein expression of MMP2, MMP9, and VE-cadherin. Thus, NR2F1 transcriptionally activates the expression of VM-related proteins, which is pivotal in promoting VM formation.

## Conclusions

The study revealed for the first time that RBMS3 and circHECTD1 both downregulated in GBM cells and tissues. circHECTD1, a newly researched HECTD1 transcriptional variant, was generated by the RNA binding protein RBMS3 through a specific interaction between RBMS3 and the flanking intron sequences of circHECTD1, thereby inhibiting VM formation in GBM. Evidence suggested that circHECTD1 encoded a functional peptide 463aa in an ORF and IRES dependent manner in the cytoplasm of GBM cells. Further, circHECTD1 and 463aa suppressed the GBM VM formation in vivo and in vitro. 463aa with low expression in GBM induced less ubiquitination modification level of transcription factor NR2F1 to increase its expression level. NR2F1 facilitates VM formation in GBM cells by transcriptionally activating the expression levels of VM-related proteins MMP2, MMP9, and VE-cadherin. In summary, our study intuitively illustrates the coding potential of circHECTD1 in VM formation in GBM. Moreover, these findings may offer valuable potential targets for anti-vascular therapy of GBM.

### Supplementary information

Supplementary figure 1

Supplementary figure 2

Supplementary figure 3

Supplementary figure 4

Supplementary figure 5

Supplementary figure 6

Supplementary figure 7

Supplementary file 1

Supplementary table 1

Supplementary table 2

aj-checklist

Original Data File

## Data Availability

All data and materials analyzed or used to support the findings in this study are available from the corresponding author upon a reasonable request.

## References

[1] Ricci-Vitiani L, Pallini R, Biffoni M, Todaro M, Invernici G, Cenci T (2010). Tumour vascularization via endothelial differentiation of glioblastoma stem-like cells. Nature..

[2] Janjua TI, Rewatkar P, Ahmed-Cox A, Saeed I, Mansfeld FM, Kulshreshtha R (2021). Frontiers in the treatment of glioblastoma: Past, present and emerging. Adv Drug Deliv Rev..

[3] Ostrom QT, Cioffi G, Gittleman H, Patil N, Waite K, Kruchko C (2019). CBTRUS Statistical Report: Primary Brain and Other Central Nervous System Tumors Diagnosed in the United States in 2012-2016. Neuro-Oncol..

[4] Ameratunga M, Pavlakis N, Wheeler H, Grant R, Simes J, Khasraw M (2018). Anti-angiogenic therapy for high-grade glioma. Cochrane Database Syst Rev..

[5] Kulikauskas MR (2022). The versatility and paradox of BMP signaling in endothelial cell behaviors and blood vessel function. Cell Mol Life Sci Cmls..

[6] Wei X, Chen Y, Jiang X, Peng M, Liu Y, Mo Y (2021). Mechanisms of vasculogenic mimicry in hypoxic tumor microenvironments. Mol Cancer..

[7] Luo Q, Wang J, Zhao W, Peng Z, Liu X, Li B (2020). Vasculogenic mimicry in carcinogenesis and clinical applications. J Hematol Oncol J Hematol Oncol..

[8] Huang XY, Huang ZL, Huang J, Xu B, Huang XY, Xu YH (2020). Exosomal circRNA-100338 promotes hepatocellular carcinoma metastasis via enhancing invasiveness and angiogenesis. J Exp Clin Cancer Res Cr..

[9] Ding J, Cui XG, Chen HJ, Sun Y, Yu WW, Luo J (2022). Targeting circDGKD Intercepts TKI’s Effects on Up-Regulation of Estrogen Receptor β and Vasculogenic Mimicry in Renal Cell Carcinoma. Cancers..

[10] Meng S, Zhou H, Feng Z, Xu Z, Tang Y, Li P (2017). CircRNA: functions and properties of a novel potential biomarker for cancer. Mol Cancer..

[11] Li X, Yang L, Chen LL (2018). The Biogenesis, Functions, and Challenges of Circular RNAs. Mol Cell..

[12] Yang Q, Li F, He AT, Yang BB (2021). Circular RNAs: Expression, localization, and therapeutic potentials. Mol Ther..

[13] Chen CK, Cheng R, Demeter J, Chen J, Weingarten-Gabbay S, Jiang L (2021). Structured elements drive extensive circular RNA translation. Mol Cell..

[14] Yang Y, Gao X, Zhang M, Yan S, Sun C, Xiao F (2018). Novel Role of FBXW7 Circular RNA in Repressing Glioma Tumorigenesis. J Natl Cancer Inst..

[15] Zheng X, Chen L, Zhou Y, Wang Q, Zheng Z, Xu B (2019). A novel protein encoded by a circular RNA circPPP1R12A promotes tumor pathogenesis and metastasis of colon cancer via Hippo-YAP signaling. Mol Cancer..

[16] Yang Y, Quan L, Ling Y (2018). RBMS3 Inhibits the Proliferation and Metastasis of Breast Cancer Cells. Oncol Res..

[17] Penkov D, Ni R, Else C, Piñol-Roma S, Ramirez F, Tanaka S (2000). Cloning of a human gene closely related to the genes coding for the c-myc single-strand binding proteins. Gene..

[18] Li Y, Chen L, Nie CJ, Zeng TT, Liu H, Mao X (2011). Downregulation of RBMS3 is associated with poor prognosis in esophageal squamous cell carcinoma. Cancer Res..

[19] Zhang T, Wu Y, Fang Z, Yan Q, Zhang S, Sun R (2016). Low expression of RBMS3 and SFRP1 are associated with poor prognosis in patients with gastric cancer. Am J Cancer Res..

[20] Wu Y, Meng D, You Y, Sun R, Yan Q, Bao J (2020). Increased expression of RBMS3 predicts a favorable prognosis in human gallbladder carcinoma. Oncol Rep..

[21] Bertacchi M, Parisot J, Studer M (2019). The pleiotropic transcriptional regulator COUP-TFI plays multiple roles in neural development and disease. Brain Res..

[22] Boudot A, Kerdivel G, Lecomte S, Flouriot G, Desille M, Godey F (2014). COUP-TFI modifies CXCL12 and CXCR4 expression by activating EGF signaling and stimulates breast cancer cell migration. BMC Cancer..

[23] Gao XL, Zheng M, Wang HF, Dai LL, Yu XH, Yang X (2019). NR2F1 contributes to cancer cell dormancy, invasion and metastasis of salivary adenoid cystic carcinoma by activating CXCL12/CXCR4 pathway. BMC Cancer..

[24] Mao XG, Xue XY, Wang L, Zhang X, Yan M, Tu YY (2013). CDH5 is specifically activated in glioblastoma stemlike cells and contributes to vasculogenic mimicry induced by hypoxia. Neuro-Oncol..

[25] Lucio-Eterovic AK, Piao Y, de Groot JF (2009). Mediators of glioblastoma resistance and invasion during antivascular endothelial growth factor therapy. Clin Cancer Res J Am Assoc Cancer Res..

[26] Seftor RE, Seftor EA, Koshikawa N, Meltzer PS, Gardner LM, Bilban M (2001). Cooperative interactions of laminin 5 gamma2 chain, matrix metalloproteinase-2, and membrane type-1-matrix/metalloproteinase are required for mimicry of embryonic vasculogenesis by aggressive melanoma. Cancer Res..

[27] Cai HP, Wang J, Xi SY, Ni XR, Chen YS, Yu YJ (2019). Tenascin-cmediated vasculogenic mimicry formation via regulation of MMP2/MMP9 in glioma. Cell Death Dis..

[28] Zhou WY, Cai ZR, Liu J, Wang DS, Ju HQ, Xu RH (2020). Circular RNA: metabolism, functions and interactions with proteins. Mol Cancer..

[29] Cai J, Chen Z, Wang J, Wang J, Chen X, Liang L (2019). circHECTD1 facilitates glutaminolysis to promote gastric cancer progression by targeting miR-1256 and activating β-catenin/c-Myc signaling. Cell Death Dis..

[30] Sinha T, Panigrahi C, Das D, Chandra, Panda A (2022). Circular RNA translation, a path to hidden proteome. Wiley Interdiscip Rev RNA..

[31] Guo B, McMillan BJ, Blacklow SC (2016). Structure and function of the Mind bomb E3 ligase in the context of Notch signal transduction. Curr Opin Struct Biol..

[32] Domsch K, Acs A, Obermeier C, Nguyen HT, Reim I (2017). Identification of the essential protein domains for Mib2 function during the development of the Drosophila larval musculature and adult flight muscles. PLoS One..

[33] Sosa MS, Parikh F, Maia AG, Estrada Y, Bosch A, Bragado P (2015). NR2F1 controls tumor cell dormancy via SOX9 and RARβ driven quiescence programs. Nat Commun..

[34] Liu Y, Zhang P, Wu Q, Fang H, Wang Y, Xiao Y (2021). Long non-coding RNA NR2F1-AS1 induces breast cancer lung metastatic dormancy by regulating NR2F1 and ΔNp63. Nat Commun..

[35] Louis C, Leclerc D, Coulouarn C (2021). Emerging roles of circular RNAs in liver cancer. JHEP Rep..

[36] Wang R, Zhang S, Chen X, Li N, Li J, Jia R (2018). EIF4A3-induced circular RNA MMP9 (circMMP9) acts as a sponge of miR-124 and promotes glioblastoma multiforme cell tumorigenesis. Mol Cancer..

[37] Lou J, Hao Y, Lin K, Lyu Y, Chen M, Wang H (2020). Circular RNA CDR1as disrupts the p53/MDM2 complex to inhibit Gliomagenesis. Mol Cancer..

[38] Dai Q, Ma Y, Xu Z, Zhang L, Yang H, Liu Q (2021). Downregulation of circular RNA HECTD1 induces neuroprotection against ischemic stroke through the microRNA-133b/TRAF3 pathway. Life Sci..

[39] Han B, Zhang Y, Zhang Y, Bai Y, Chen X, Huang R (2018). Novel insight into circular RNA HECTD1 in astrocyte activation via autophagy by targeting MIR142-TIPARP: implications for cerebral ischemic stroke. Autophagy..

[40] Li C, Liu Y, Lv Z, Zheng H, Li Z, Zhang J (2021). Circular RNA circHECTD1 facilitates glioma progression by regulating the miR-296-3p/SLC10A7 axis. J Cell Physiol..

[41] Li W, Wang S, Shan B, Cheng X, He H, Qin J (2021). CircHECTD1 Regulates Cell Proliferation and Migration by the miR-320-5p/SLC2A1 Axis in Glioblastoma Multiform. Front Oncol..

[42] Górnicki T, Lambrinow J, Mrozowska M, Podhorska-Okołów M, Dzięgiel P, Grzegrzółka J (2022). Role of RBMS3 Novel Potential Regulator of the EMT Phenomenon in Physiological and Pathological Processes. Int J Mol Sci..

[43] Zhu L, Xi PW, Li XX, Sun X, Zhou WB, Xia TS (2019). The RNA binding protein RBMS3 inhibits the metastasis of breast cancer by regulating Twist1 expression. J Exp Clin Cancer Res Cr..

[44] Chen J, Kwong DLW, Zhu CL, Chen LL, Dong SS, Zhang LY (2012). RBMS3 at 3p24 Inhibits Nasopharyngeal Carcinoma Development via Inhibiting Cell Proliferation, Angiogenesis, and Inducing Apoptosis. PLoS One..

[45] Wu G, Cao L, Zhu J, Tan Z, Tang M, Li Z (2019). Loss of RBMS3 Confers Platinum Resistance in Epithelial Ovarian Cancer via Activation of miR-126-5p/β-catenin/CBP signaling. Clin Cancer Res J Am Assoc Cancer Res..

[46] Patop IL, Wüst S (2019). Kadener S. Past, present, and future of circRNAs. EMBO J..

[47] Conn SJ, Pillman KA, Toubia J, Conn VM, Salmanidis M, Phillips CA (2015). The RNA binding protein quaking regulates formation of circRNAs. Cell..

[48] Dong W, Dai ZH, Liu FC, Guo XG, Ge CM, Ding J (2019). The RNA-binding protein RBM3 promotes cell proliferation in hepatocellular carcinoma by regulating circular RNA SCD-circRNA 2 production. EBioMedicine..

[49] Yu CY, Li TC, Wu YY, Yeh CH, Chiang W, Chuang CY (2017). The circular RNA circBIRC6 participates in the molecular circuitry controlling human pluripotency. Nat Commun..

[50] Sun J, Li B, Shu C, Ma Q, Wang J (2020). Functions and clinical significance of circular RNAs in glioma. Mol Cancer..

[51] Sun W, Zhou H, Han X, Hou L, Xue X (2021). Circular RNA: A novel type of biomarker for glioma. Mol Med Rep..

[52] Wu P, Mo Y, Peng M, Tang T, Zhong Y, Deng X (2020). Emerging role of tumor-related functional peptides encoded by lncRNA and circRNA. Mol Cancer..

[53] Li LJ, Leng RX, Fan YG, Pan HF, Ye DQ (2017). Translation of noncoding RNAs: Focus on lncRNAs, pri-miRNAs, and circRNAs. Exp Cell Res..

[54] Ma S, Kong S, Wang F, Ju S (2020). CircRNAs: biogenesis, functions, and role in drug-resistant Tumours. Mol Cancer..

[55] Liang WC, Wong CW, Liang PP, Shi M, Cao Y, Rao ST (2019). Translation of the circular RNA circβ-catenin promotes liver cancer cell growth through activation of the Wnt pathway. Genome Biol..

[56] Zhang M, Huang N, Yang X, Luo J, Yan S, Xiao F (2018). A novel protein encoded by the circular form of the SHPRH gene suppresses glioma tumorigenesis. Oncogene..

[57] Wang Y, Wu C, Du Y, Li Z, Li M, Hou P (2022). Expanding uncapped translation and emerging function of circular RNA in carcinomas and noncarcinomas. Mol Cancer..

[58] Bernassola F, Chillemi G, Melino G (2019). HECT-Type E3 Ubiquitin Ligases in Cancer. Trends Biochem Sci..

[59] Pan Z, Zhao R, Li B, Qi Y, Qiu W, Guo Q (2022). EWSR1-induced circNEIL3 promotes glioma progression and exosome-mediated macrophage immunosuppressive polarization via stabilizing IGF2BP3. Mol Cancer..

[60] Zhao Y, Song J, Dong W, Liu X, Yang C, Wang D (2022). The MBNL1/circNTRK2/PAX5 pathway regulates aerobic glycolysis in glioblastoma cells by encoding a novel protein NTRK2-243aa. Cell Death Dis..

