# Supplementary material for: RBMS3-induced circHECTD1 encoded a novel protein to suppress the vasculogenic mimicry formation in glioblastoma multiforme
Source: Cell Death Dis. 2023 Nov 15;14(11):745. doi: 10.1038/s41419-023-06269-y (PMC10651854; doi:10.1038/s41419-023-06269-y)
Supplement: Supplementary file 1 — Supplementary figure 1 [file 41419_2023_6269_MOESM1_ESM.docx]

Supplementary figure 1


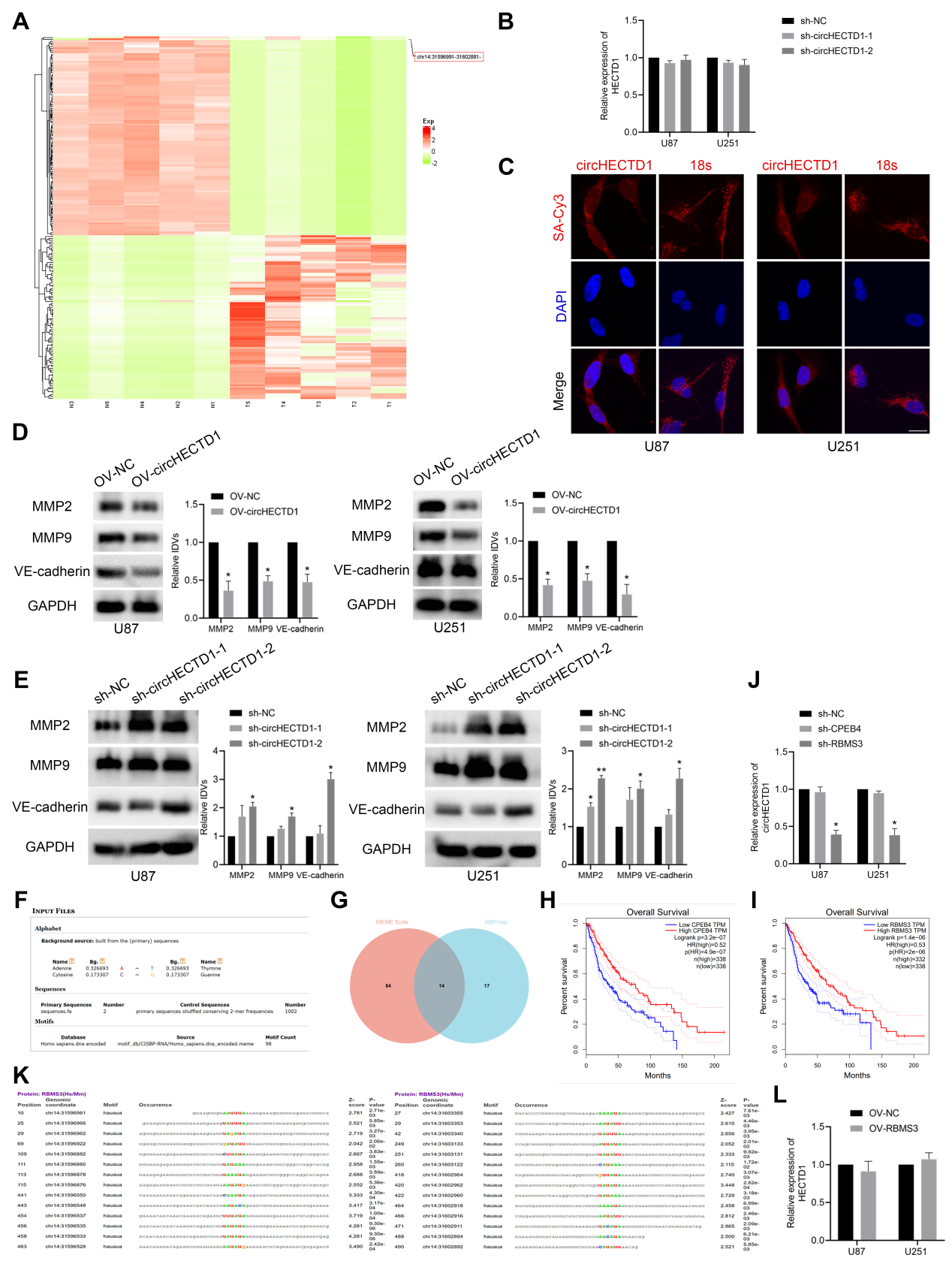


Supplementary figure 1. Screening of circHECTD1 and RBMS3 in GBM.

1. The analysis of hsa_circ_0002301 expression in 5 normal brain tissues and 5 GBM tissues in GSE92322. (**B**) Relative expression of HECTD1 mRNA in U87 and U251 cells after circHECTD1 knockdown (n=3). (**C**) The subcellular localization of SA-Cy3-labeled circHECTD1 was shown by RNA FISH assay, with 18s rRNA as a positive control. Scale bar=10μm. (**D**) Relative expression of MMP2, MMP9, and VE-cadherin in U87 and U251 cells with circHECTD1 elevation was tested by western blot. (n=3), **P*<0.05 vs. OV-NC group. (**E**) Relative expression of VM-related protein in GBM cells with circHECTD1 knockdown was shown. (n=3), **P*<0.05 vs. sh-NC group. (**F**) MEME suite was applied to analyze circHECTD1 flanking intron sequences. (**G**) MEME suite and RBPmap were applied to predict the RBPs that bind to both 500bp flanking intron sequences upstream and downstream of the circHECTD1 junction site, respectively. (**H**-**I**) Effect of CPEB4 and RBMS3 expression on GBM and LGG patient survival time from GEPIA database. (**J**) Relative expression of circHECTD1 after CPEB4 or RBMS3 knockdown. (n=3), **P*<0.05 vs. sh-NC group. (**K**) Predicted binding sites between RBMS3 and circHECTD1 flanking intron sequences (±500bp) using RBPmap database. (**L**) Relative expression of HECTD1 mRNA in U87 and U251 cells with RBMS3 overexpression (n=3).
