# Supplementary material for: RBMS3-induced circHECTD1 encoded a novel protein to suppress the vasculogenic mimicry formation in glioblastoma multiforme
Source: Cell Death Dis. 2023 Nov 15;14(11):745. doi: 10.1038/s41419-023-06269-y (PMC10651854; doi:10.1038/s41419-023-06269-y)
Supplement: Supplementary file 2 — Supplementary figure 2 [file 41419_2023_6269_MOESM2_ESM.docx]

Supplementary figure 2


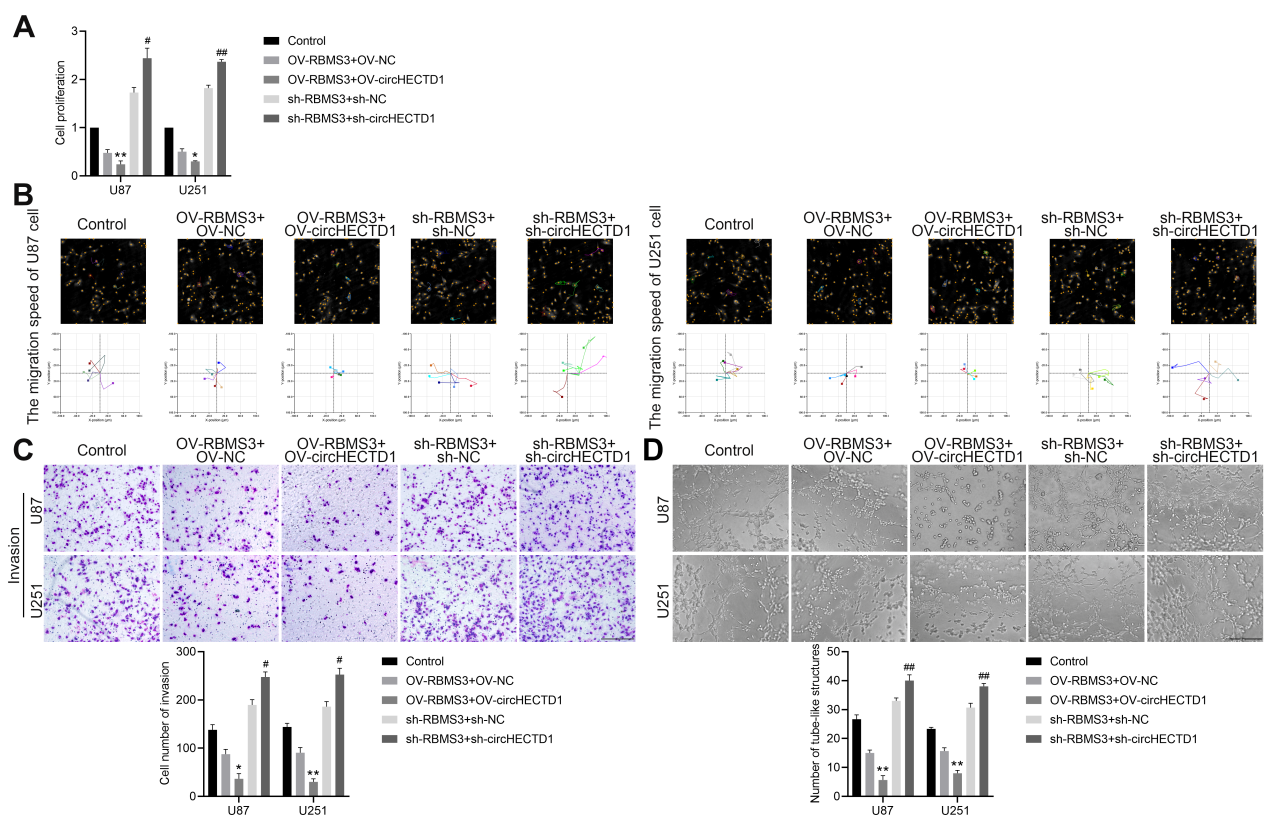


Supplementary figure 2. Effects of bidirectional treatment with RBMS3 and circHECTD1 on VM formation in GBM cells.

(**A**) The CCK8 assay was applied to detect changes in the proliferative capacity of U87 and U251 cells. (**B**) The digital holographic microscopy was applied to detect changes in the migrative capacity of U87 and U251 cells. (**C**) The transwell assay was applied to detect changes in the invasive capacity of U87 and U251 cells. (**D**) The in vitro tube formation assay was applied to detect changes in the tube-formed capacity of U87 and U251 cells. (n=3). **P*<0.05, ***P*<0.01 vs. OV-RBMS3+OV-NC group; ^#^*P*<0.05, ^##^*P*<0.01 vs. sh-RBMS3+sh-NC group. Scale bar, 200μm.
