# Supplementary material for: RBMS3-induced circHECTD1 encoded a novel protein to suppress the vasculogenic mimicry formation in glioblastoma multiforme
Source: Cell Death Dis. 2023 Nov 15;14(11):745. doi: 10.1038/s41419-023-06269-y (PMC10651854; doi:10.1038/s41419-023-06269-y)
Supplement: Supplementary file 3 — Supplementary figure 3 [file 41419_2023_6269_MOESM3_ESM.docx]

Supplementary figure 3


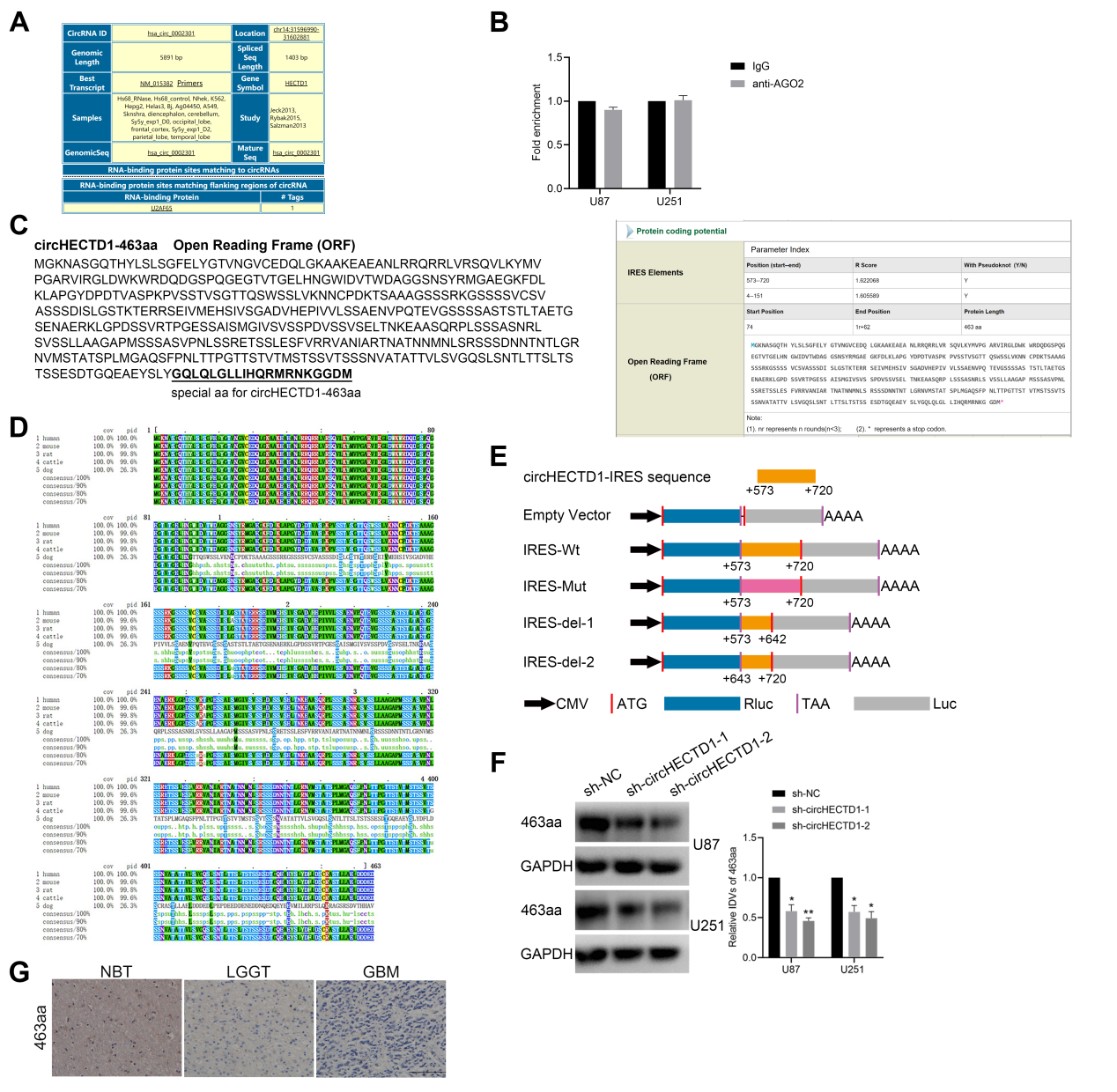


Supplementary figure 3. circHECTD1 had the ability to encode polypeptide.

(**A**) CircInteractome website showed the potentially binding RBPs with corcHECTD1. (**B**) RIP analysis of AGO2 binding to circHECTD1 in U87 and U251 cells (n=3). (**C**) TheThe analysis of circHECTD1 in circRNADb database and the identified the special aa for circHECTD1-463aa. (**D**) Conservation analysis of the putative ORF in circHECTD1 by Clustal Omega website. (**E**) The illustration of IRES sequences in circHECTD1 or its different truncations or mutation were cloned between Rluc and Luc reporter genes with independent start and stop codons. (**F**) Relative expression of 463aa in U87 and U251 cells with circHECTD1 knockdown was shown (n=3). **P*<0.05, ***P*<0.01 vs. sh-NC group. (**G**) The 463aa expression in NBT, LGGT, and GBM was shown by the IHC assay. Scale bar=200μm.
