# Supplementary material for: RBMS3-induced circHECTD1 encoded a novel protein to suppress the vasculogenic mimicry formation in glioblastoma multiforme
Source: Cell Death Dis. 2023 Nov 15;14(11):745. doi: 10.1038/s41419-023-06269-y (PMC10651854; doi:10.1038/s41419-023-06269-y)
Supplement: Supplementary file 4 — Supplementary figure 4 [file 41419_2023_6269_MOESM4_ESM.docx]

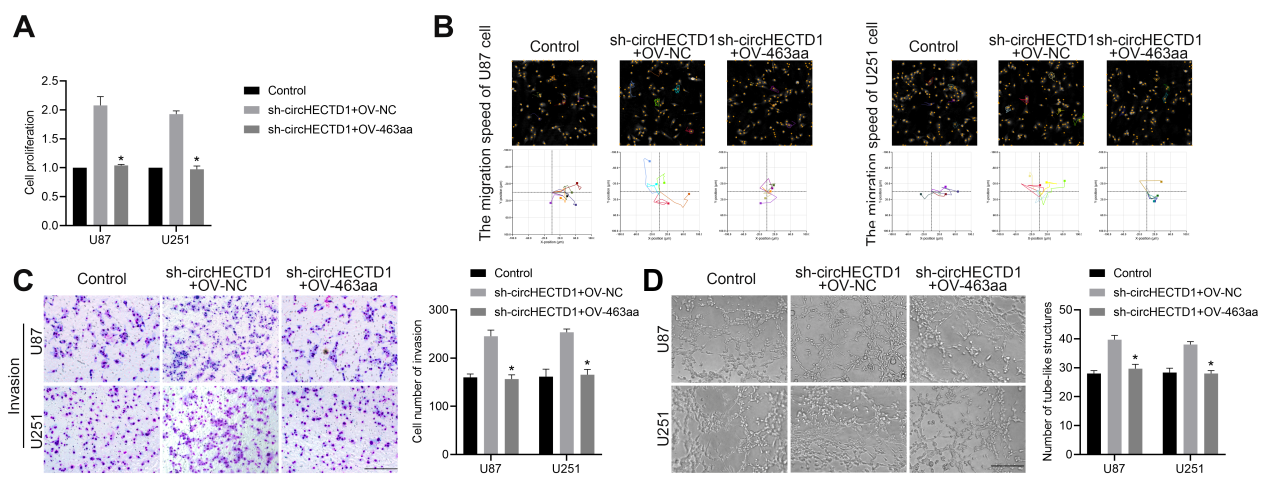
Supplementary figure 4

Supplementary figure 4. Effect of 463aa overexpression based on silencing circHECTD1 on VM formation in GBM cells.

(**A**) Upregulated 463aa on the basis of circHECTD1 knockdown in U87 and U251 cells. The CCK8 assay was applied to detect changes in the proliferative capacity. (**B**) The digital holographic microscopy was applied to detect changes in the migrative capacity. (**C**) The transwell assay was applied to detect changes in the invasive capacity. (**D**) The in vitro tube formation assay was applied to detect changes in the tube-formed capacity (n=3). **P*<0.05 vs. sh-circHECTD1+OV-NC group. Scale bar=200μm.
