# Supplementary material for: RBMS3-induced circHECTD1 encoded a novel protein to suppress the vasculogenic mimicry formation in glioblastoma multiforme
Source: Cell Death Dis. 2023 Nov 15;14(11):745. doi: 10.1038/s41419-023-06269-y (PMC10651854; doi:10.1038/s41419-023-06269-y)
Supplement: Supplementary file 5 — Supplementary figure 5 [file 41419_2023_6269_MOESM5_ESM.docx]

Supplementary figure 5


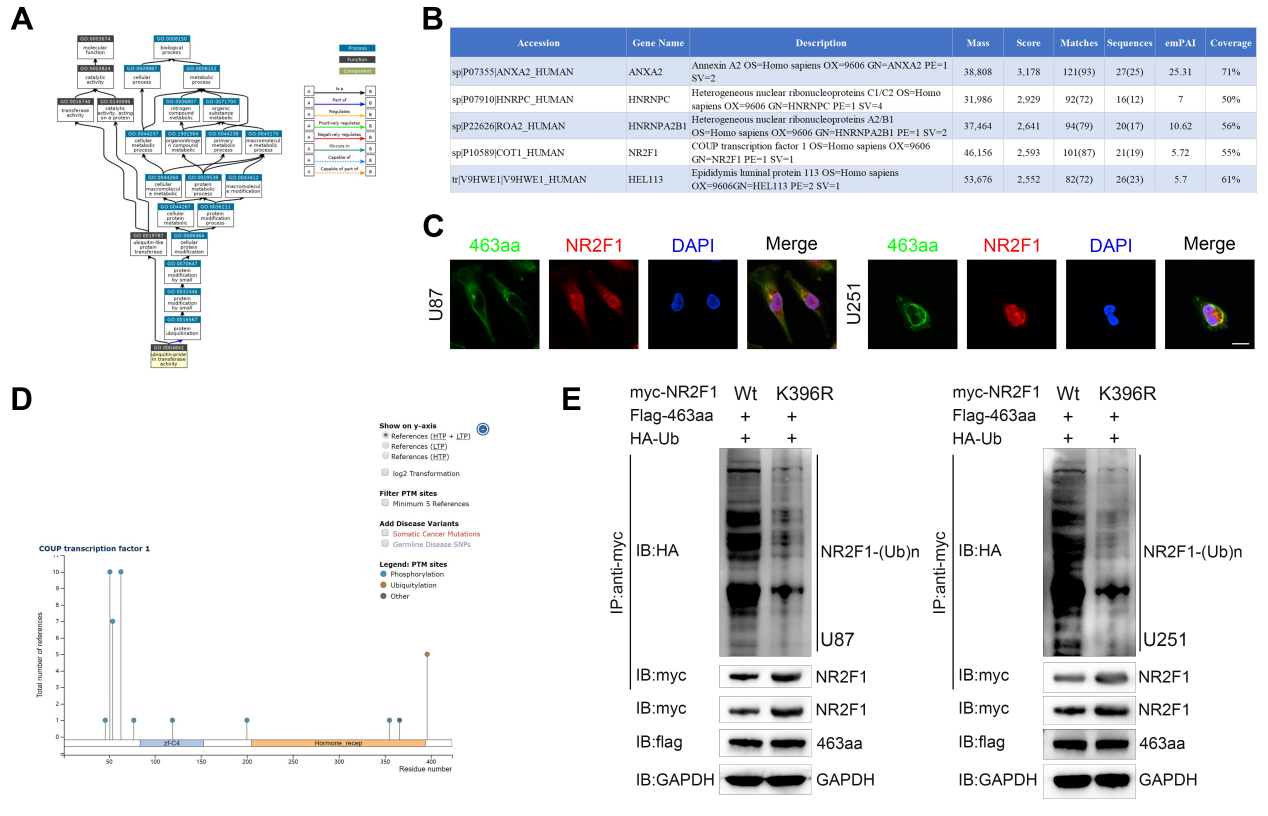


Supplementary figure 5. 463aa interacted with NR2F1 in GBM cells.

(**A**) The GO analysis of 463aa molecular function by InterPro database. (**B**) 463aa interacting proteins screened by MS analysis of the precipitates. (**C**) Co-localization of 463aa (green) with NR2F1 (red) in U87 and U251 cells was shown by IF assay. Scale bar=10μm. (**D**) The putative ubiquitination site of NR2F1 was shown by the Uniprot database. (**E**) Polyubiquitination of NR2F1-Wt or its mutant was tested by transfection with indicated plasmids in GBM cells followed by co-immunoprecipitation analysis.
