# Supplementary material for: RBMS3-induced circHECTD1 encoded a novel protein to suppress the vasculogenic mimicry formation in glioblastoma multiforme
Source: Cell Death Dis. 2023 Nov 15;14(11):745. doi: 10.1038/s41419-023-06269-y (PMC10651854; doi:10.1038/s41419-023-06269-y)
Supplement: Supplementary file 6 — Supplementary figure 6 [file 41419_2023_6269_MOESM6_ESM.docx]

Supplementary figure 6


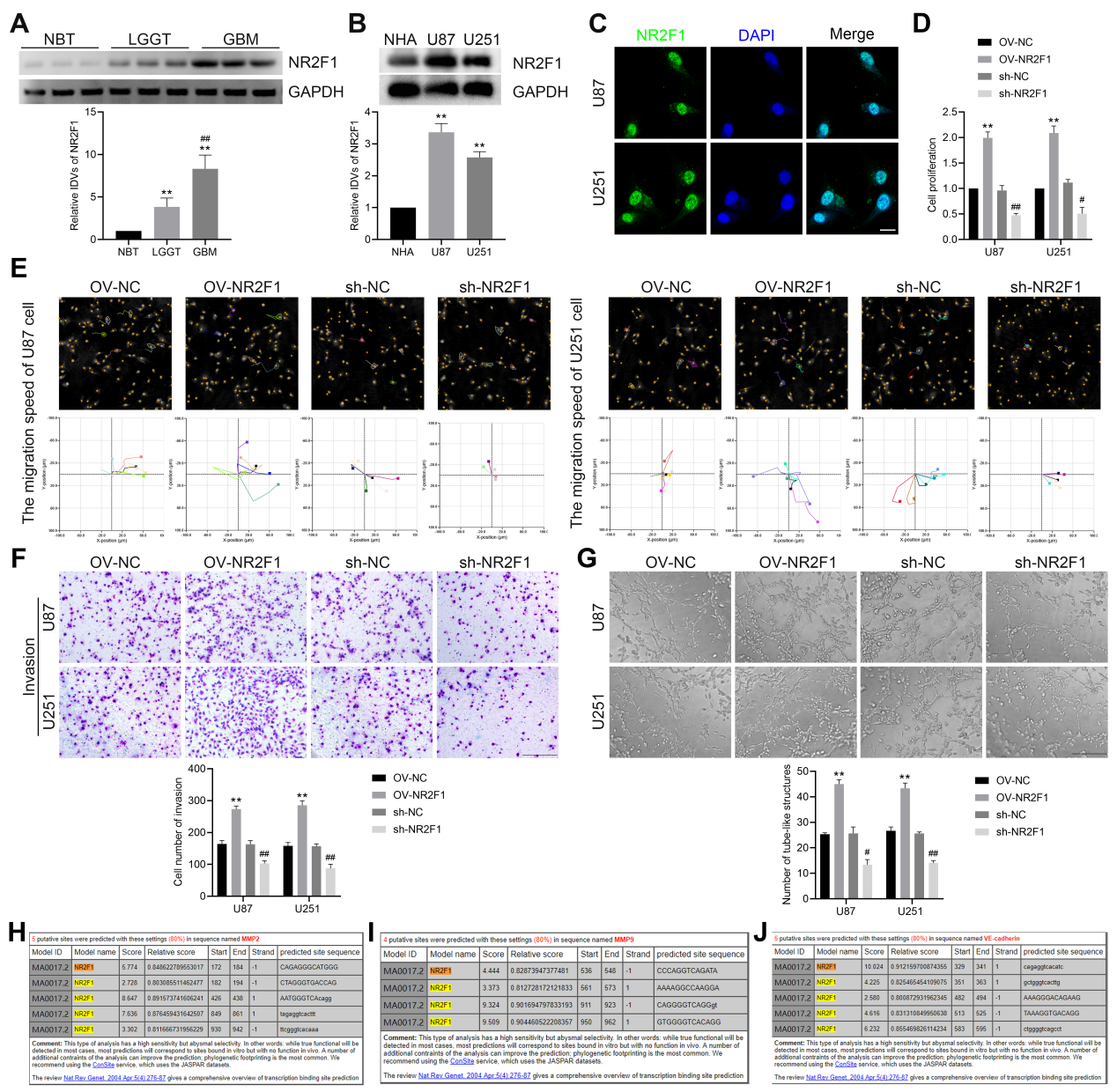


Supplementary figure 6. Knockdown of NR2F1 inhibited the VM formation in GBM cells.

(**A**) Relative expression of NR2F1 in NBT (n=3), LGGT (n=9), and GBM (n=9) was shown by western blot. ***P*<0.01 vs. NBT group; ^##^*P*<0.01 vs. LGGT group. (**B**) The expression level of NR2F1 in NHA, U87, and U251 cells (n=3). ***P*<0.01 vs. NHA group. (**C**) Subcellular distribution of NR2F1 in U87 and U251 cells shown by IF assay. Scale bar=10μm. (**D-G**) Overexpression of NR2F1 promoted, while knockdown inhibited the cell proliferation, migration, invasion, and VM-channel formation of U87 and U251 cells (n=3). ***P*<0.01 vs. OV-NC group; ^#^*P*<0.05, ^##^*P*<0.01 vs. sh-NC group. Scale bar=200μm. (**H-J**) The JASPAR database predicted the binding sites of NR2F1 to the promoter regions of MMP2, MMP9 and VE-cadherin, respectively.
