# Supplementary material for: RBMS3-induced circHECTD1 encoded a novel protein to suppress the vasculogenic mimicry formation in glioblastoma multiforme
Source: Cell Death Dis. 2023 Nov 15;14(11):745. doi: 10.1038/s41419-023-06269-y (PMC10651854; doi:10.1038/s41419-023-06269-y)
Supplement: Supplementary file 7 — Supplementary figure 7 [file 41419_2023_6269_MOESM7_ESM.docx]

Supplementary figure 7


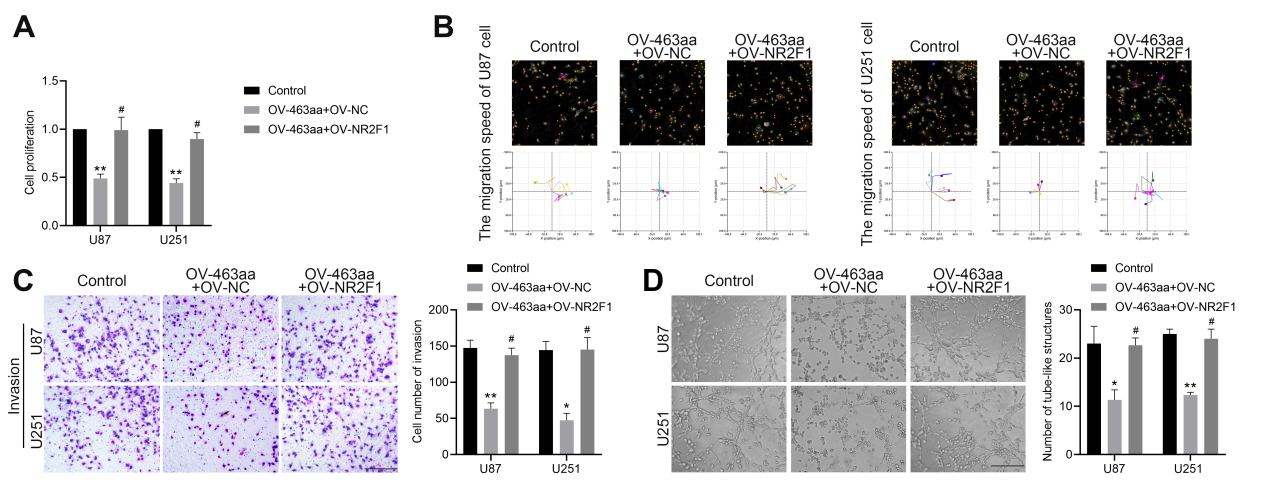


Supplementary figure 7. Effect of NR2F1 overexpression on VM formation in GBM cells based on 463aa upregulation.

(**A**) The CCK8 assay was applied to detect changes in the proliferative capacity of U87 and U251 cells. (**B**) The digital holographic microscopy was applied to detect changes in the migrative capacity. (**C**) The transwell assay was applied to detect changes in the invasive capacity. (**D**) The in vitro tube formation assay was applied to detect changes in the tube-formed capacity (n=3). **P*<0.05, ***P*<0.01 vs. Control group; ^#^*P*<0.05 vs. OV-463aa+OV-NC group. Scale bar=200μm.
