# Supplementary material for: RBMS3-induced circHECTD1 encoded a novel protein to suppress the vasculogenic mimicry formation in glioblastoma multiforme
Source: Cell Death Dis. 2023 Nov 15;14(11):745. doi: 10.1038/s41419-023-06269-y (PMC10651854; doi:10.1038/s41419-023-06269-y)
Supplement: Supplementary file 8 — Supplementary file 1 [file 41419_2023_6269_MOESM8_ESM.docx]

**Materials and Methods**

**Cell Culture and Human Tissue Samples**

GBM cell lines (U87 and U251) and HEK293T cells were purchased from the Shanghai Institutes for Biological Sciences Cell Resource Center. And these cells were cultured in DMEM high glucose medium with 10% fetal bovine serum (FBS) (Gibco, USA) added. Normal human astrocytes (NHA) were obtained from Shanghai Zeye Biotechnology and cultured in a complete growth medium. All cells were cultured in stable 37°C humidified incubators with 5% CO_2_. In this study, nine samples of low-grade and GBM tissues were used, and those were classified by neuropathologists according to 2016 WHO classification. Normal brain tissues (NBTs) and glioma tissues were obtained from hospitalized patients who signed the informed consent form in the Department of Neurosurgery of Shengjing Hospital of China Medical University. Moreover, our study was approved by the Ethics Committee of China Medical University.

**Antibodies**

The primary antibodies are as follows: RBMS3 (Cat# A17142, RRID: AB_2771963, ABclonal Technology), NR2F1 (Cat# 24573-1-AP, RRID: AB_2879616, Proteintech Group), MMP2 (Cat#10373-2-AP, RRID: AB_2250823, Proteintech Group), MMP9 (Cat# 10375-2-AP, RRID: AB_10897178, Proteintech Group), VE-cadherin (Cat# 66804-1-Ig, RRID: AB_ 2882147, Proteintech Group), ubiquitin (Cat# 10201-2-AP, RRID: AB_671515, Proteintech Group), Flag (Cat# F1804, Sigma-Aldrich), Myc (Cat# sc-40, Santa Cruz Biotechnology), HA (Cat# sc-805, Santa Cruz Biotechnology) and GAPDH (Cat# 60004-1-Ig, RRID: AB_2107436, Proteintech Group).

**Cell Transfection**

The short hairpin RNAs against circHECTD1 (sh-circHECTD1: site #1, 5’-GAATATTCCTTATATGGTCAA-3', site #2, 5’-CCTTATATGGTCAACTGCAAC-3') and NR2F1 (sh-NR2F1: 5’-CGTCCGCAGGAACTTAACTTA-3') were synthesized by GenePharma, and their corresponding empty plasmids (sh-circHECTD1-NC and sh-NR2F1) were constructed as the negative control. The plasmids with RBMS3 full-length sequence (OV-RBMS3), circHECTD1 full-length sequence (OV-circHECTD1), circHECTD1-463aa full-length sequence (OV-463aa), NR2F1 full-length sequence (OV-NR2F1), NR2F1 with wild-type K396 (NR2F1-WT) or mutant K396 (NR2F1-K396R), and their corresponding empty plasmids were also constructed. The renilla luciferase (Rluc) and the firefly luciferase (Luc) sequences were amplified from a psicheck2 vector. The Rluc was at the front, and the Luc was in the back. The full-length Rluc-Luc sequences were acquired using overlapping PCR, and the flank sequences were connected to the pCDNA3.1(+) vector via two restriction enzyme sites, NheI and XhoI. The putative IRES sequences of circHECTD1 were amplified and placed between Rluc and Luc by two restriction enzyme sites, kpnI and EcoRI, introduced by primers. Lipofectamine 3000 (Invitrogen, USA) was used to transfect the plasmids according to the manufacturer's instructions.

**Immunoprecipitation and immunoblot analysis**

10min of incubation at 4°C with lysis solution containing protease inhibitors (Sigma, USA) was sufficient to lyse the cells. Following centrifugation, protein concentrations were determined, and equal volumes of lysates were utilized for immunoprecipitation. The lysates were immunoprecipitated using antibodies and protein A-Sepharose (Santa Cruz Biotechnology, USA) overnight at 4°C. Immune complexes were then eluted with sample buffer containing 1% SDS for 5min at 95°C and examined by SDS-PAGE. Using secondary antibodies coupled to horseradish peroxidase and chemiluminescence, immunoblotting was conducted.

**Cell Migration assay**

The migration capacity of GBM cells was observed by the digital holographic microscopy in the HoloMonitor M4 culture system (Phase Holographic Imaging PHI AB, SE) in vitro. The cells of separate groups were planted at a concentration of 2×10^4^ cells/mL into a six-well plate. After the cells were attached to the petri dish, they were placed on the HoloMonitor M4 culture system and imaged for 6h at 1h intervals. For each experimental group, the final visual frame and cell motions were displayed. At the beginning of the investigation, five visually identifiable cells from each trial set were chosen for tracking. Their movements were represented by X-Y plots in space.

**Transwell assay**

Glioma cells that had been digested were planted into polycarbonate membrane chambers at a number of around 20,000 cells per well. DMEM high-glucose medium without FBS was used in the upper chamber, while high-glucose medium with 10% FBS was used in the lower chamber. Matrigel was introduced to the polycarbonate membrane chamber before the cell invasiveness test. The cells were removed from the culture after 24h, and a microscope was used to evaluate the cell migration ability.

**In Vitro Tube Formation assay**

100μL Matrigel Basement Membrane Matrix (BD Biosciences, USA) was added to each well of the 96-well culture plate. At 37°C, the 96-well culture plate was incubated for 30min. The cells were resuspended in 100μL of serum-free media, plated at a density of 6×10^5^ cells/mL on Matrigel, and incubated for 4h. Under an inverted microscope (Olympus, Japan), the cellular vasculature was seen and photographed. An independent observer determined the number of tube-like structures in each photograph.

**Tumor xenografts in nude mice**

Lentivirus encoding circHECTD1 and circHECTD1-463aa was produced with the pLenti6.3/V5eDEST Gateway Vector Kit (Life Technologies, USA). To establish stably expressing cells of Lv-circHECTD1 and Lv-circHECTD1-463aa, target lentivirus vectors were constructed. Beijing Vital River Laboratory Animal Technology Co., Ltd. (Beijing, China) supplied four-week-old BALb/C athymic nude mice. The mice were separated into five groups: Control, Lv-vector, Lv-circHECTD1, Lv-circHECTD1-463aa, and Lv-circHECTD1+Lv-circHECTD1-463aa. 3×10^5^ (100μL) cells were subcutaneously injected into the right limb of nude mice. We observed and recorded the tumor formation time and measured the weight and volume of the transplanted tumor to generate a tumor growth curve [tumor volume (mm^3^) = (longest diameter × shortest diameter)^2^×0.5]. For survival investigation, 3×10^5^ (10μL) cells were stereotactically transplanted into the right striatum of nude mice. The number of surviving nude mice was recorded, and a Kaplan–Meier survival analysis was conducted.
