# Supplementary material for: RBMS3-induced circHECTD1 encoded a novel protein to suppress the vasculogenic mimicry formation in glioblastoma multiforme
Source: Cell Death Dis. 2023 Nov 15;14(11):745. doi: 10.1038/s41419-023-06269-y (PMC10651854; doi:10.1038/s41419-023-06269-y)
Supplement: Supplementary file 9 — Supplementary table 1 [file 41419_2023_6269_MOESM9_ESM.docx]

Supplementary table 1

The primer sequence of circHECTD1, NR2F1, MMP2, MMP9, VE-cadherin, HECTD1, GAPDH and U6 used in quantitative real-time PCR was shown.

|  | **Forward primers** | **Reverse primers** |
| --- | --- | --- |
| circHECTD1 | ACCAGCCTCACATCAACTTCCA | GTGGATCAAGAGGCCAAGTTGC |
| NR2F1 | TTCGTCCGTTTGGTAGGTAAAA | GAGCACTGGATGGACATGTAAG |
| MMP2 | CACAGCAGGTCTCAGCCTCATC | GCCAAGCGGTCTAAGTCCAGAG |
| MMP9 | TGGTCCTGGTGCTCCTGGTG | TGCCTGTCGGTGAGATTGGTTC |
| VE-cadherin | GAGACTCCTTCCAGCTTCACCATC | ACTCAGGCACGGACGCATTG |
| HECTD1 | CCTTGCACTCTGCTATGGCT | CAGAGCTCCATCTGAAACCTGA |
| GAPDH | GGTGAAGGTCGGAGTCAACG | CCATGTAGTTGAGGTCAATGAAG |
| U6 | CGCAAGGATGACACGCAA | GTGCAGGGTCCGAGGT |
