# Supplementary material for: RBMS3-induced circHECTD1 encoded a novel protein to suppress the vasculogenic mimicry formation in glioblastoma multiforme
Source: Cell Death Dis. 2023 Nov 15;14(11):745. doi: 10.1038/s41419-023-06269-y (PMC10651854; doi:10.1038/s41419-023-06269-y)
Supplement: Supplementary file 10 — Supplementary table 2 [file 41419_2023_6269_MOESM10_ESM.docx]

Supplementary table 2

|  | **Forward primers** | **Reverse primers** | **Product size** |
| --- | --- | --- | --- |
| MMP2-Control | CGCACTATACGAGGCCAAGT | TCAGAAACAGATGATCAGCCACA | 201bp |
| MMP2-binding site | TTCAAGGATCTGGAGCTGGC | ctctgttggctcgctcttga | 207bp |
| MMP9-Control | Ttcattcaatttctccccatgctg | GGGTCTAAAACTTTTATCAGATACTCA | 201bp |
| MMP9-binding site | CTTTCTCATGCTGGTGCTGC | CACCAGGAGCACCAGGAC | 213bp |
| VE-cadherin-Control | agGAGTCCCAAGGAGAGCTT | caagccatccatcctgggag | 203bp |
| VE-cadherin-binding site | Tggctccaggatgacacaac | CCTCGGTCTGTAGGGGATGT | 105bp |
