# Supplementary figures and images for: RBMS3-induced circHECTD1 encoded a novel protein to suppress the vasculogenic mimicry formation in glioblastoma multiforme
Source: Cell Death Dis. 2023 Nov 15;14(11):745. doi: 10.1038/s41419-023-06269-y (PMC10651854; doi:10.1038/s41419-023-06269-y)

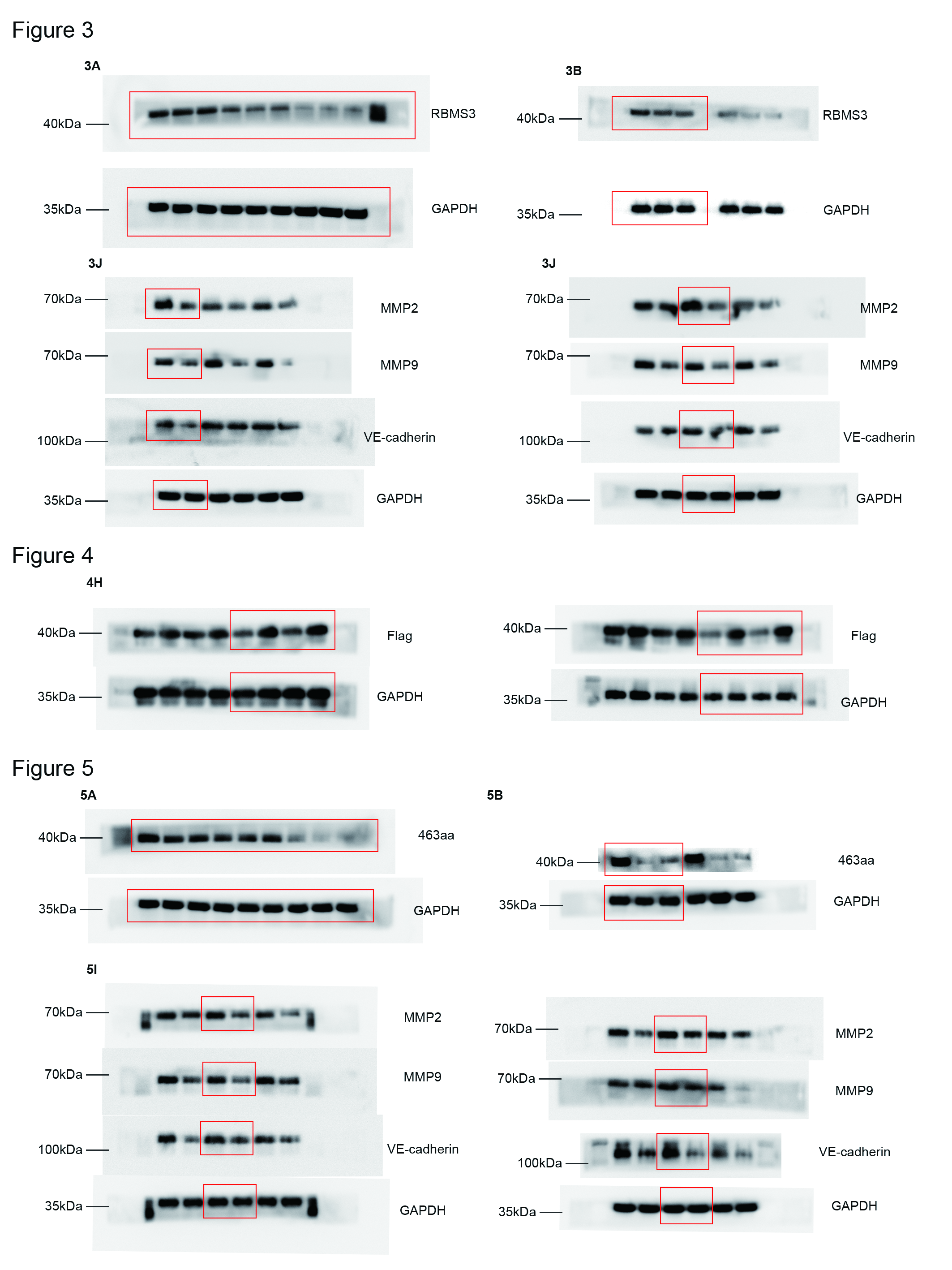


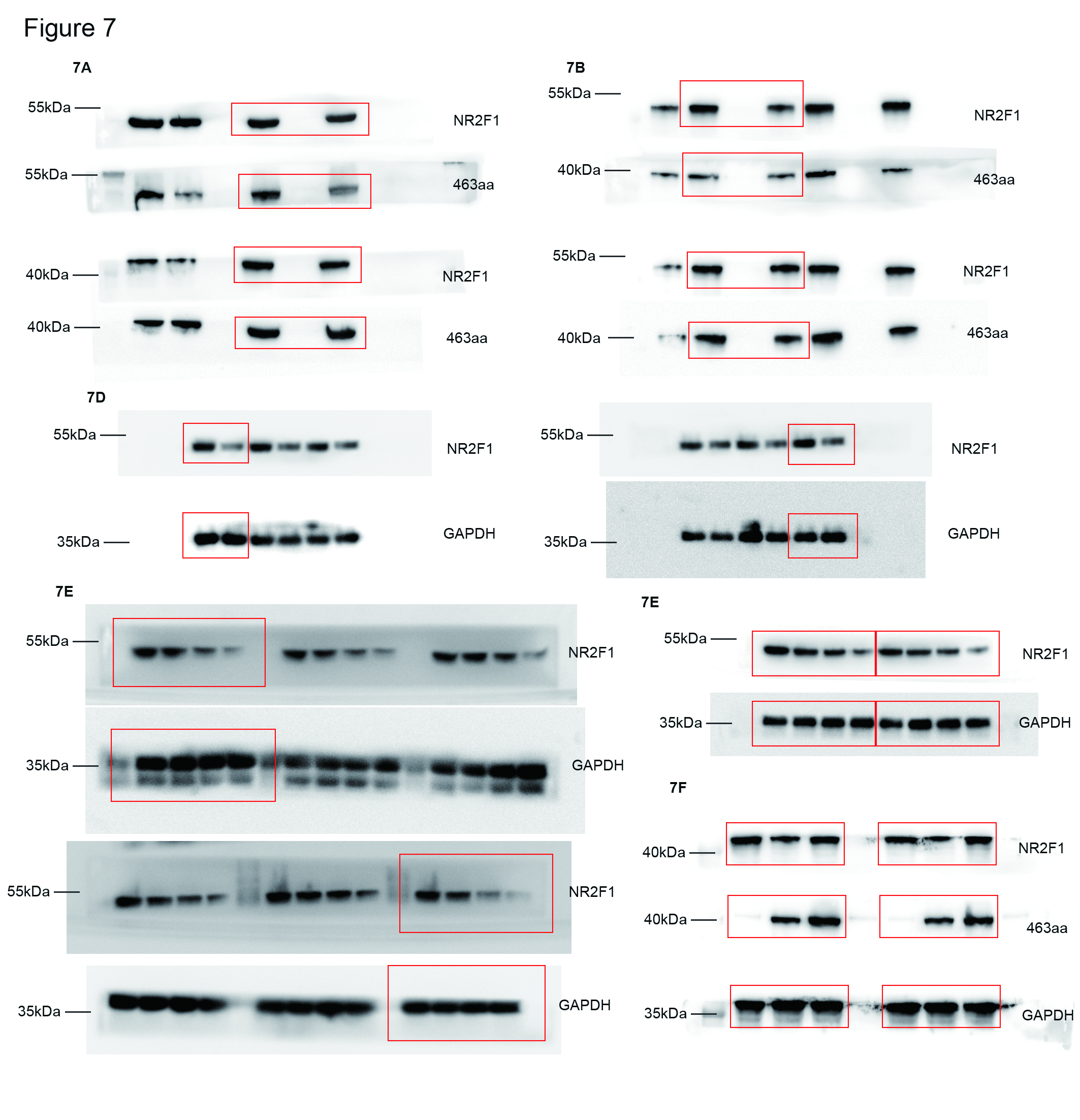


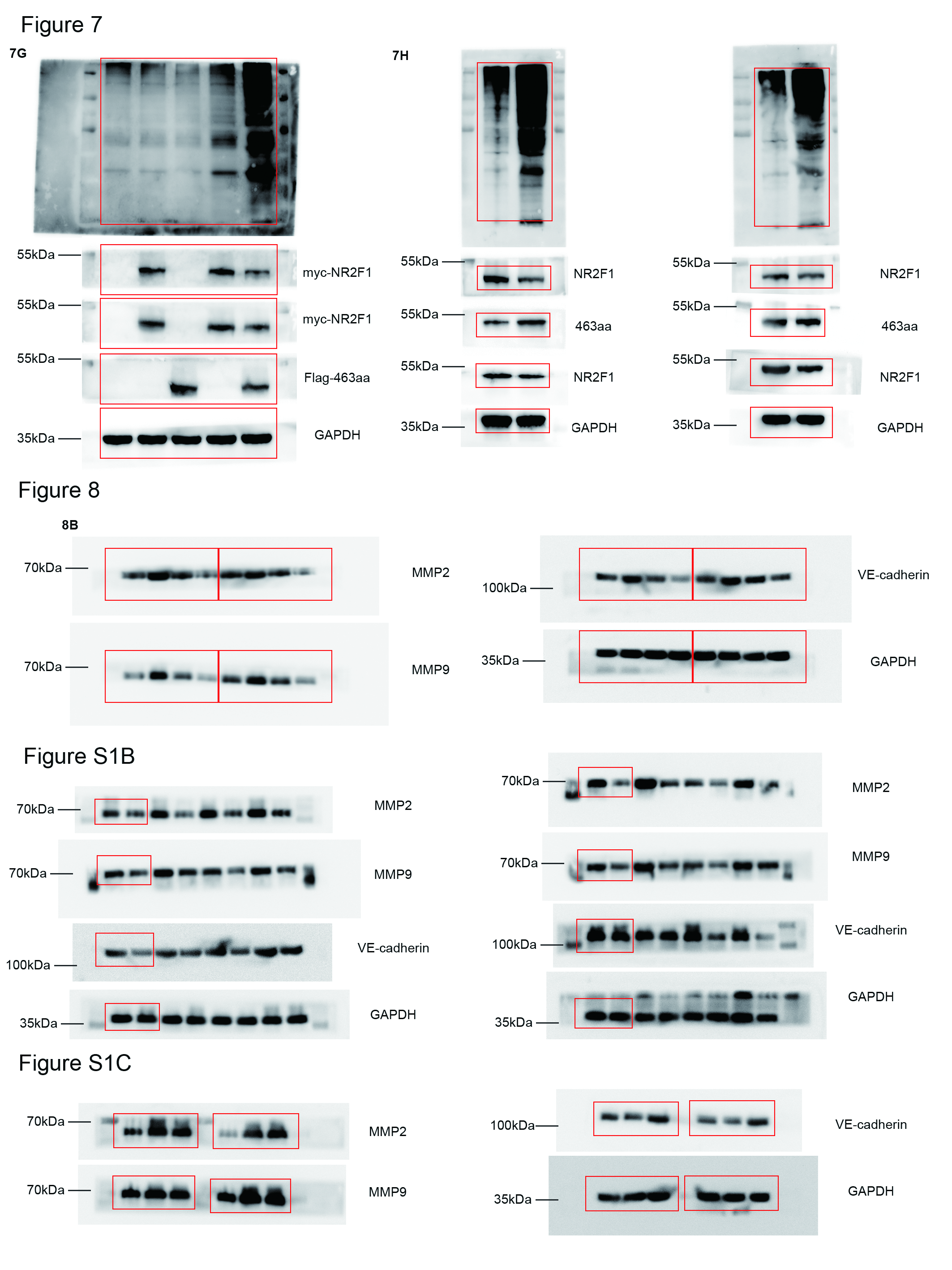


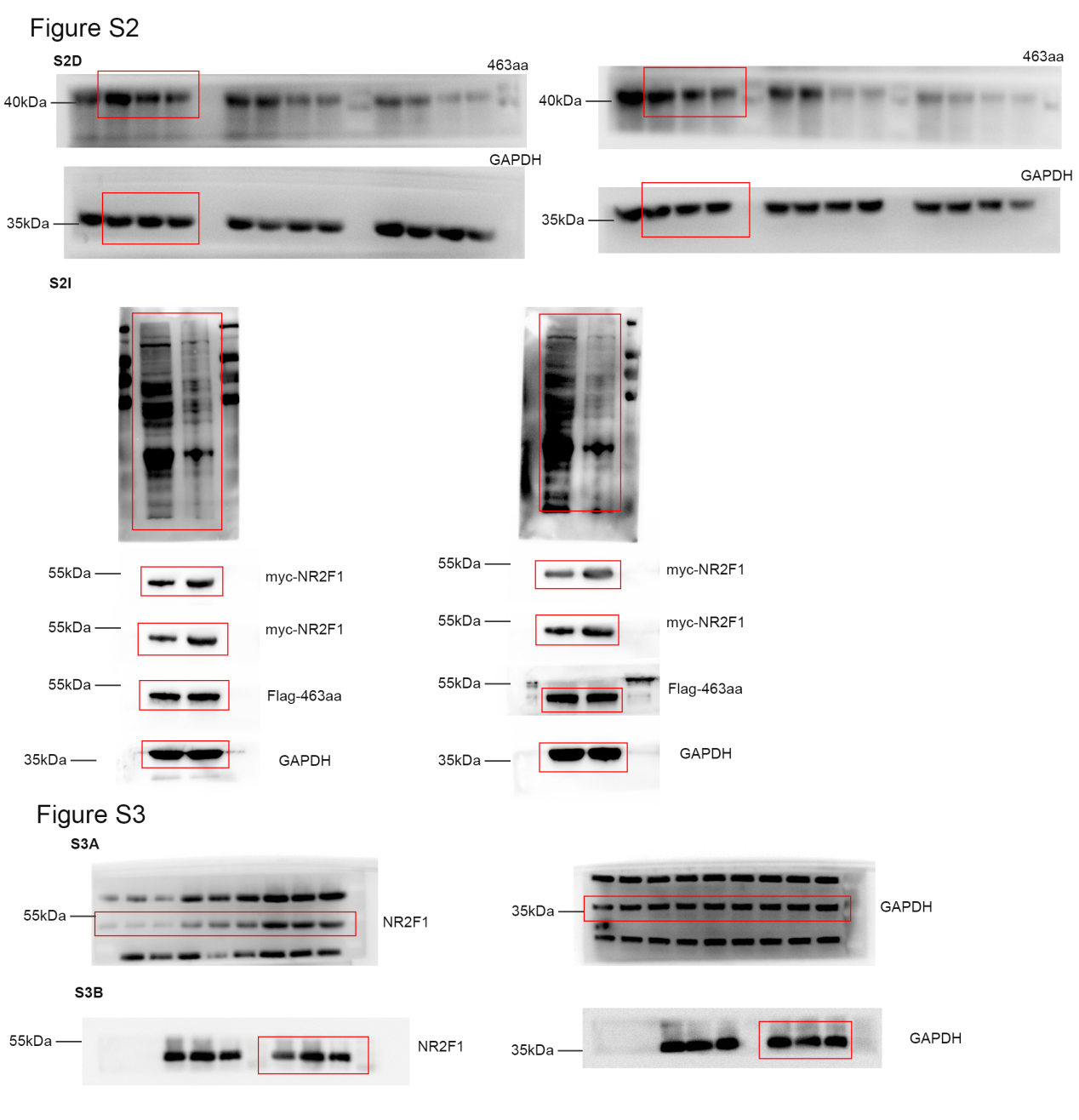

Supplement: Supplementary file 12 — Original Data File [file 41419_2023_6269_MOESM12_ESM.docx]
